# In Situ Formation of Zeolitic Imidazolate Frameworks on Nanocellulose Revealed by Time-Resolved Synchrotron Small-Angle and Wide-Angle X‑ray Scattering

**DOI:** 10.1021/acsami.5c10734

**Published:** 2025-08-18

**Authors:** Salvatore Lombardo, Houssine Khalili, Shun Yu, Sritama Mukherjee, Kim Nygård, Zoltan Bacsik, Aji P. Mathew

**Affiliations:** † Department of Chemistry, Stockholm University, 10691 Stockholm, Sweden; ‡ Unit of Material, Surface and Barrier, Department of Sustainable Materials and Packaging, 388792 RISE Research Institute of Sweden, 22363 Lund, Sweden; § Department of Fiber and Polymer Technology, School of Chemistry, Biotechnology and Health, KTH Royal Institute of Technology, 10044 Stockholm, Sweden; ∥ MAX IV Laboratory, 5193 Lund University, 22100 Lund, Sweden; ⊥ Institute of Chemistry, 61764 University of Miskolc, 3515 Miskolc, Hungary

**Keywords:** ZIF-8, nanocellulose, CelloZif-8, kinetics of particle growth, mechanism of formation, X-ray scattering

## Abstract

Metal–organic frameworks such as ZIF-8, grown in situ on nanocellulose (NC), have gained significant attention in recent years due to the versatility of the processing route and multifaceted application in the field of environmental remediation and biomedical applications. However, insights into the interactions between NC and MOF precursors and MOF structure evolution during in situ synthesis are limited or nonexistent. We report the kinetics of ZIF-8 formation on a nanocellulose (NC) aqueous suspension and in water at room temperature, monitored in real time after the addition of ZIF-8 precursors. This is the first study revealing the mechanism of ZIF-8 formation in the presence of nanocellulose. A combination of synchrotron-based small-angle (SAXS) and wide-angle X-ray scattering (WAXS) enabled us to compare the time evolution of the radius of gyration obtained from SAXS and the extent of crystallization determined by WAXS. Based on the SAXS data, we propose a new model that accounts for the initial rapid formation of primary particles, which subsequently evolve into medium-range structures before growing into the final product. Scanning electron microscopy images supported this mechanism, showing smaller particles at the beginning of the reaction and confirmed interparticle interactions, showing nanocellulose particles decorating the surface of the final ZIF-8 crystals. We demonstrate that the concentration of the starting metal salt significantly influences the kinetics of the reaction but has little effect on the ZIF-8 particle size. In contrast, increasing the NC concentration led to a reduction in the final ZIF-8 particle size, while having a negligible impact on the reaction rate and affording a minor decrease in surface area and micropore volume. We show that at the lower NC concentration that was studied the ZIF-8 particles were covered by NC, and no reduction in porosity was observed. Moreover, the kinetics of formation was shown to be independent of the NC functional group and morphology under the conditions used in this study.

## Graphical abstract

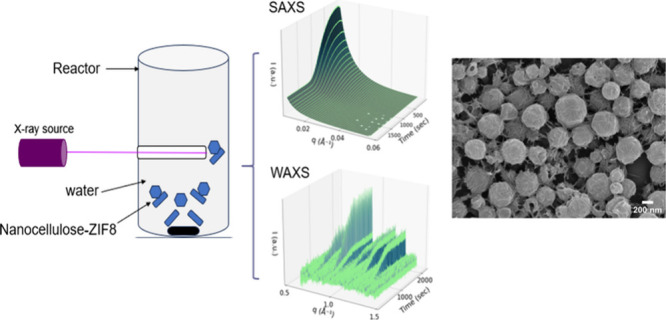

## 1 Introduction

In recent years, research on zeolitic imidazolate frameworks (ZIFs) has attracted significant attention for several applications, such as gas capture,^1^ water/oil separation,^2^ water purification,^3^ catalysis,^4^ etc. ZIFs are a subclass of metal–organic frameworks (MOFs) and possess excellent properties, such as chemical and structural stability, well-defined and tunable porosity, and a large surface area. ZIF-8 is a popular and typical member of this family, featuring a 2-methylimidazolate linker tetrahedrally coordinating metallic zinc ions forming a sodalite topology and large pores with a diameter of 11.6 Å, which can be accessed through small apertures with a diameter of 3.4 Å.^5,6^ ZIF-8 presents several advantages, such as high selective separation, robustness (Young’s modulus ∼ 3–4 GPa), and BET surface areas that reach 1950 m^2^ g^–1^. ZIF-8 can be synthesized in different solvents using different methods, showing different particle size distributions under different experimental conditions, while the specific surface area remains in the same range.^7^

ZIF-8 particles form through an Ostwald ripening process, where at equilibrium larger particles are thermodynamically favored, leading to the dissolution of smaller particles.^8^ Previous studies of the kinetics of ZIF-8 formation were reported under various experimental conditions, such as using methanol as a solvent,^9,10^ or using mechanochemical,^11^ solvothermal,^12,13^ and sonocrystallization synthesis.^14^ In the mentioned works, WAXS was used as the main technique to determine the kinetics of formation, by following the appearance and evolution of the crystallization peaks typical of ZIF-8. These works used either the Avrami–Erofeev (AE) model or the Gualtieri model^15^ to fit the data. Cravillon et al. used a combination of SAXS and WAXS to study the formation of ZIF-8 in methanol.^16^ Moh et al. used atomic force microscopy (AFM) to study the structural changes occurring during the synthesis of ZIF-8 in methanol, showing that ZIF-8 growth occurs simultaneously through a “birth-and-spread” and a spiral growth mechanism in different regions of the crystal face.^17^ An approach based on machine learning was recently proposed to study the mechanism of ZIF-8 formation, highlighting a complex interplay between synthetic variables.^18^ Pan et al. reported the first successful synthesis of ZIF-8 in water at room temperature, which is more convenient compared to organic solvents, as it is cheaper and nontoxic.^19^ Liu et al. proposed a three-step mechanism for the formation of ZIF-8 in water, using cryo-TEM and electron diffraction, showing a liquid–liquid phase separation in the first step to form solute-rich and solute-poor regions, which condense in a second step to form an amorphous aggregate and then crystallize into ZIF-8.^20^

ZIF-8 has been utilized in combination with support materials to make composites.^21^ Supported ZIF-8 presents several advantages. For example, it may weaken the tendency of the particles to aggregate, thus increasing the surface area.^22^ It can increase the water solubility of ZIF-8 and make it easier to process into 3D macroscopic materials. For instance, hybrid materials prepared combining ZIF-8 and other MOFs with cellulose nanomaterials (CelloZIF-8) have been the subject of a recent review.^23^ Nanocellulose provides a biodegradable, renewable, and highly functionalized platform, while ZIF-8 contributes to high porosity, tunable structures, and high adsorption capacities. CelloZIF-8 displays enhanced mechanical stability and improved processability compared to those of ZIF-8. For instance, nanocellulose/ZIF-8 hybrids can be processed into membranes,^23^ nanopapers, aerogels,^24^ foams,^25^ and 3D printing materials.^1,21,26^ Such hybrid materials show significant promise for applications in gas separation, heavy metal ion adsorption, drug delivery, and catalysis, where their properties offer a clear edge.^23^

Indeed, CelloZIF-8 displays notable properties for practical applications. For instance, CelloZIF-8 aerogels showed an adsorption capacity of up to 558.66 mg/g for Pb­(II) ions and 1060.2 mg/g for malachite green. In addition, these materials showed a CO_2_ adsorption capacity of 1.63 mmol/g with a CO_2_/N_2_ selectivity of 22, highlighting their potential for gas separation.^23^

Such hybrid materials can be prepared by the ex situ blending method, where ZIF-8 is synthesized and then mixed with cellulose nanomaterial in water, or by the in situ method, where the synthesis of ZIF-8 is performed in the presence of nanocellulose.^23^ It was reported that the in situ preparation strategy leads to stronger interfacial bonding between the nanocellulose substrate and the MOF.^27^

However, the synthesis strategies for CelloZIF-8 and more generally for CelloMOFs are not yet fully optimized; the fundamental understanding of the interactions stabilizing the structure and the reaction mechanism is still incomplete, and it is unclear how the presence of nanocellulose influences the mechanism of the formation of ZIF-8.

In this work, we studied the kinetics of the formation of ZIF-8 in water and in a nanocellulose aqueous suspension for the first time, using a combination of SAXS and WAXS, in operando.

The main advantage of combining SAXS and WAXS is the high brilliance of the synchrotron radiation used, which enabled a low time resolution, and the possibility of obtaining detailed information about particle size and their crystalline properties simultaneously. This method was successfully used to study the kinetics of formation of ZIF-8 and other metal–organic frameworks,^16,28,29^ but never in combination with nanocellulose or other support materials. SAXS is sensitive to the particle size and shape in the nanometer range and can detect amorphous or noncrystalline particles that appear during early nucleation, whereas WAXS provides structural information about crystalline phases. Together, they offer a continuous view from the formation of very small precursors to the growth of fully developed crystals, enabling the study of both noncrystalline intermediates and crystalline final products within the same experiment.^8^

In our experiments, we extended the use of combined SAXS and WAXS beyond traditional diluted suspensions to study particle growth in a dense nanocellulose matrix, which itself scatters strongly at small angles and presents challenges due to its large structural size relative to the SAXS resolution. Our method provided highly reproducible measurements, which allowed us to successfully follow the growth of ZIF-8 particles significantly larger than those typically accessible by SAXS alone. By cross-validating SAXS results with WAXS, we confirmed consistent trends between the two techniques, demonstrating the robustness and innovative nature of our approach in a complex and highly scattering environment. We studied the influence of nanocellulose surface functionalities, morphologies, and concentrations of both reagents and nanocellulose on the crystallization process. Based on these measurements, we proposed a mechanism for the formation of CelloZIF-8 in water. Understanding the mechanism of nucleation and growth, as well as the structural changes associated with CelloMOF formation, is crucial to achieve better control of structure formation.

## 2 Experimental Section

### 2.1 Materials

Sulfated cellulose nanocrystals (SCNCs) were purchased from Celluforce. Phosphorylated cellulose nanofibers (PCNFs) and Tempo-oxidized cellulose nanofibers (TOCNFs) were prepared and characterized in our lab following our previously reported procedures.^30^ In brief, SCNC has a surface coverage of 0.15–0.28 mmol of sulfate esters g^–1^, as specified by the producer, PCNF 3 mmol g^–1^, and TOCNF 1 mmol g^–1^. 2-Methylimidazole (Hmim, 99%), zinc nitrate hexahydrate (98%), and triethylamine (TEA) were purchased from Sigma-Aldrich.

### 2.2 Synthesis of CelloZIF-8

ZIF-8 and CelloZIF-8 were synthesized in water using a previously reported three-step procedure.^3^ In the first step, a solution of Zn­(NO_3_)_2_ (2–15 mM) was added to 15 mL of either pure water or a nanocellulose suspension (0.02 or 0.2 wt %) at neutral pH. In the second step of the synthesis, a solution of TEA was added (2–15 mM). The molar concentration of TEA was the same as the Zn­(NO_3_)_2_ concentration. In the last step, an excess of organic linker was added (0.3–1 M). A list of experiments and detailed synthesis conditions is provided in Table S1. The sample was washed twice by centrifugation with a water/ethanol mixture and two subsequent times with water (10 000 rpm, 10 min) before characterization.

### 2.3 In Situ Synchrotron-Based SAXS/WAXS Measurements

We used time-resolved simultaneous small- and wide-angle X-ray scattering (SAXS/WAXS) at the ForMAX beamline,^31^ MAX IV laboratory, to follow the formation of ZIF-8 particles in water in the absence and presence of nanocellulose. The X-ray energy was 16.4 keV with sample-to-detector distances of ∼2.5 m for the SAXS detector (Eiger2 × 4M, Detectric Ltd.) and 211.1 mm for the WAXS detector (Lambda 3M, X-spectrum GmbH). The experimental configuration can cover *q* ranges from 0.1 to 2.0 nm^–1^ for the SAXS detector and from 8.0 to 30.0 nm^–1^ for the WAXS detector, which collects several sharp Bragg reflections of ZIF-8. We used a customer-made quartz beaker reactor with a nose cone pointing inward, with a solution path of ∼1.0 mm along the beam path, which allowed the probe X-ray to be probed continuously while magnetically stirring (see Figure S1).

### 2.4 Wide-Angle X-ray Scattering Data

Wide-angle X-ray scattering data were normalized by dividing the scattering intensity by their Porod invariant *Q*:

1 $$Q=\int_{q_{0}}^{q_{n}}4\pi I\left(q\right)q^{2}\;dq$$

2 $$I{\left(q\right)}_{n}=I\left(q\right)/Q$$

The background signal from the beaker and nanocellulose was subtracted after normalization. We applied a custom-made baseline to the data. The Avrami model (AE) was used to fit the time-resolved data, according to the following equation:^32^

3 $$\mathrm{crystallization extent}=1-e^{-k_{a}t^{n}}$$

where *k*_a_ is the Avrami constant and *n* is the Avrami exponent.

### 2.5 Small-Angle X-ray Scattering Data

A rectangular parallelepiped with uniform scattering length density model^33^ was used to interpret the scattering signal of SCNC and that after the addition of Zn^2+^, which had been previously used for nanocellulose suspensions.^34^ SAXS data obtained after the addition of the organic linker were fitted use the Guiner–Porod model.^35^ Fitting was performed using SasView 5.0.6 (www.sasview.org) and a custom-built python script based on the same equations. The model combines two regimes: one at lower scattering vectors (*q*), where the scattering intensity decreases exponentially (Guiner region) as a function of *q*, and the other at higher scattering vectors, where the scattering intensity follows a power law decay (Porod region). If we consider *q*_1_ as the split point of the region, the model can be expressed as follows:

4 $$I\left(q\right)=\{\begin{array}{l} \frac{1}{q^{s}}e^{-{\frac{{R_{g}}^{2}q}{3}}^{2}}q<q_{1}\\ \frac{D}{q^{m}}q>q_{1} \end{array}$$

where *R*_g_ is the radius of gyration of the particle, *s* is a dimensionality parameter related to the shape of the particles, and *m* is the Porod exponent related to the surface characteristics.

The transition point between the Guinier and Porod regions, *q*_1_, is given by

5 $$q_{1}=\frac{1}{R_{g}}\sqrt{\frac{\left(m-s\right)\left(3-s\right)}{2}}$$

Parameter *D* is derived from the continuity of the Guinier and Porod functions and is related to the amplitude of the scattering when *q* > *q*_1_. It can be expressed as

6 $$D=\frac{1}{R_{g}}e^{\left(-\frac{m-s}{2}\right){\left(\frac{\left(m-s\right)\left(3-s\right)}{2}\right)}^{\left(m-s\right)/2}}$$

To model the time dependency of *R*_g_ determined from the Guiner–Porod model, we first used an equation based on Finke–Watzky two-step model for particle formation:^36^

7 $$R_{g}\left(t\right)=R_{1}+\left(R_{f}-R_{1}\right){\left(1-\frac{k_{1}+k_{2}\left[P\right]}{k_{2}\left[P\right]+k_{1}e^{k_{1}+k_{2}\left[P\right]t}}\right)}^{1/3}$$

where *R*_1_ is the initial radius of gyration, corresponding to the initial radius of the starting nuclei, and *R*_f_ is the radius of the final particles.

A three-step model was used to account for the starting exponential increase of primary particles and was adapted from this model, adding an initial empirical exponential step where primary particles grow exponentially.

8 $$R_{g}\left(t\right)=\{\begin{array}{ll} R_{1}\left[1-{e^{-k_{1}\left(t\right)}}^{1/3}\right] & t<t_{1}\\ f\times R_{1}+\left(R_{f}-f\times R_{1}\right){\left(1-\frac{k_{2}+k_{3}\left[P\right]}{k_{3}\left[P\right]+k_{1}e^{k_{2}+k_{3}\left[P\right]t}}\right)}^{1/3} & t>t_{1} \end{array}$$

To decrease the number of fitting parameters, the values of *k*_1_ and *R*_1_ were fitted separately, and *t*_1_ was taken at the point when the correlation coefficients at *t* > *t*_1_ were the highest. A factor *f* was used to correlate the two fits, which was always in the range between 0.97 and 1.

### 2.6 Morphological Characterization

Scanning electron microscopy (SEM) was used to determine the morphology of the ZIF-8 and CelloZIF-8 structures, using JEOL IT800 SEM equipment, operating at 5 kV. The samples were previously coated with a thin layer of gold. Atomic force microscopy (AFM) was conducted for imaging of the nanocellulose using a Dimension FastScan instrument. Suspensions were diluted to 0.001 wt % and subjected to sonication prior to the imaging.

### 2.7 Gas Adsorption Data

N_2_ isotherms were measured with a Micromeritics ASAP2020 instrument at 77 K. Prior to the measurements, the samples were activated at 378 K for 10 h in dynamic vacuum (<1 Pa). The surface areas of the materials were determined by the BET model from the adsorption branch of the N_2_ isotherms. The points for the calculation of the surface area were selected applying the Rouquerol criteria; typically, a few points were selected below relative pressures of 0.06 that fulfilled the criteria. The t-plot model was used to calculate the external surface areas of the samples. In the absence of a very appropriate density functional theory (DFT) model, the pore size distribution was determined with a model that considered spherical/cylindrical pores. This model was employed only to compare the similarity and difference in the porosity of the samples and not to characterize the porosity of the materials fully accurately.

### 2.8 Powder X-ray Diffraction

X-ray diffraction of powder products was performed using a D8 Discover X-ray Powder Diffractometer from Bruker. Scans were performed in the *q* range from 0.7 to 5.2, for 10 min.

## 3 Results and Discussion

CelloMOFs are hybrid functional materials prepared by combining metal–organic frameworks and cellulose. Due to their porous structure, CelloMOFs are promising as adsorbent materials for water and air remediation. Mathew and co-workers have developed a methodology to grow porous zeolitic imidazolate frameworks (ZIF-8) on a cellulose matrix (CelloZIF) to design hybrids with intriguing functions such as pollutant adsorption, CO_2_ capture, controlled drug release, electrochemical sensing, etc.^1,26^ While the mechanism of nucleation and growth of unsupported ZIF-8 was studied by time-resolved SAXS/WAXS,^16^ the mechanism of formation of ZIF-8 on cellulose supports is still unexplored.

### 3.1 Synthesis of CelloZIF-8 and ZIF-8 in Water

In this study, simultaneous SAXS/WAXS was used to determine the mechanism of ZIF-8 formation in water with and without nanocellulose. We used SCNCs (0.2 wt %) as the main model system as they formed a stable suspension at that concentration. We also used TOCNFs and PCNFs. At a concentration of 0.2 wt %, both TOCNFs and PCNFs formed a gel and could not be used for scattering studies, as gelation would prevent adequate stirring, thus leading to radiation damage. A lower concentration of 0.02 wt % was used for cellulose nanofibers. An overview of the suspensions used and their morphological characterization by AFM is shown in Figure S2. Time-resolved SAXS provided insights into the structural changes occurring during ZIF-8 synthesis, and the appearance of crystallization peaks in the WAXS region indicated the progressive growth of ZIF-8 crystals.

We studied all three steps of the methodology described in section 2.2 using SAXS/WAXS. In the first preliminary step, we added a Zn­(NO_3_)_2_ solution, and we reported the effect of nanocellulose complexation by Zn^2+^. In a second preliminary step, we added TEA to the suspension, which led to the formation of a precipitate. In the final step, we added the organic linker, which led to the formation of ZIF-8 particles. While the WAXS profile did not show any changes (Figure S3) until the appearance of the ZIF-8 crystallization peaks, changes in the SAXS scattering profiles were measured in every stage of the full methodology. An overview of the changes recorded in the full methodology by SAXS is given in Figure 1.

**Figure 1:**
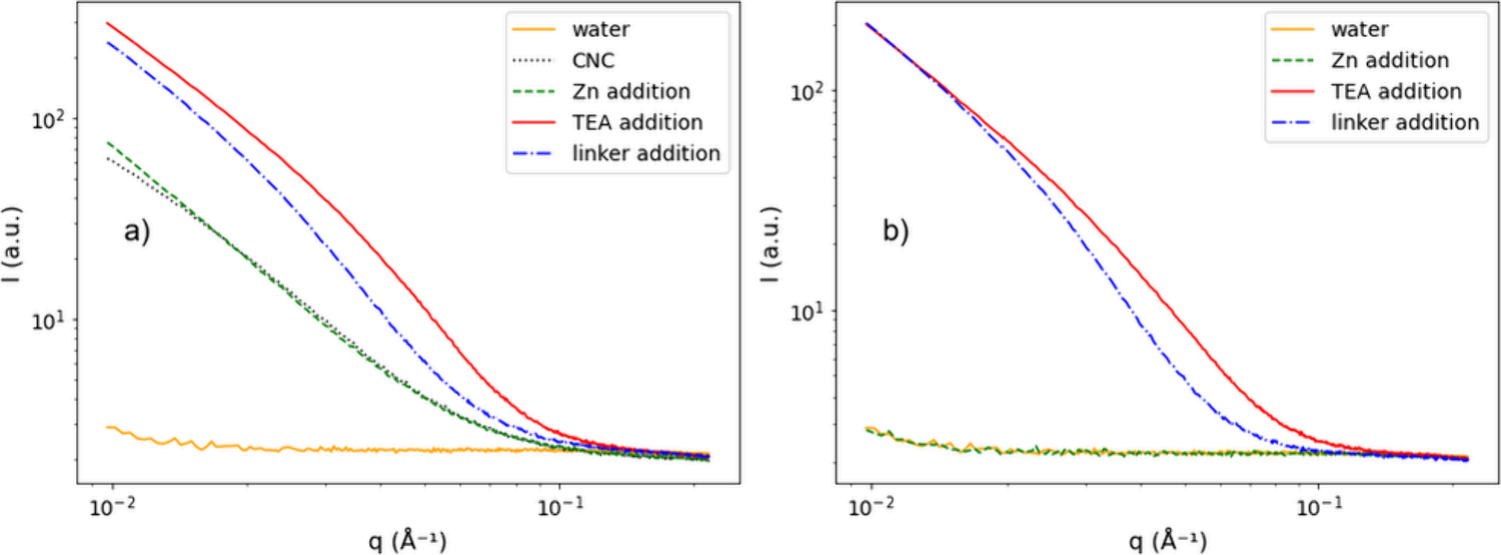
Example of SAXS scattering patterns measured during the full methodological study. (a) Synthesis of SCNC/ZIF-8 and (b) synthesis of ZIF-8 in water. (a) Water (orange solid line), addition of a 0.2% SCNC suspension (black dotted line), addition of 9 mM Zn­(NO_3_)_2_ (green dashed line), addition of 9 mM TEA (red solid line), and addition of 0.6 M Hmim (blue dashed–dotted line). (b) Water (orange solid line), addition of 9 mM Zn­(NO_3_)_2_ (green dashed line), addition of 9 mM TEA (red solid line), and addition of 0.6 M Hmim (blue dashed–dotted line).

In the first step, nanocellulose was mixed with a Zn­(NO_3_)_2_ solution. The WAXS profile did not show any change (Figure S3), while the SAXS scattering profile slightly changed (see Figure 1). No changes were recorded for a pure Zn­(NO_3_)_2_ solution (see Figure 1b). We determined the form factor using a rectangular prism model, and the results were comparable to literature values for wood SCNCs.^37^ The values relative to the length of the cross section increased linearly after the addition of Zn^2+^ (see Figure S4b). A previous study of cellulose nanocrystal (CNC) nanopaper complexation by positive ions was reported by Paul et al., using anomalous small-angle X-ray scattering. They reported that the addition of metal ions leads to an increase in size, which was associated with the formation of a Stern layer.^38^ In our work, we found a comparable trend using SCNCs in suspension. SAXS studies of Zn^2+^ and Cu^2+^ complexation of TOCNF suspensions and films were reported in a previous work from our group, showing a re-entrant phase transition of the TOCNF suspension that was dependent on the ion concentration, and Cu­(II) reduction to Cu­(I).^39,40^ We studied the thermodynamics of interactions and cation-induced gelation of negatively charged CNCs, showing that phase transition started when the CNC surface groups were saturated with counterions.^41,42^ In this work, we used different conditions as both SCNCs were 10 times more diluted compared to literature conditions and they did not form a gel phase. Also, no reduction of Zn­(II) was observed, which is expected due to the different redox behavior compared to that with Cu­(II).

In the second part of our methodology, we added TEA to precipitate the Zn^2+^ ions, which led to the formation of a ZnO precipitate, as reported by the previous work of Abdelhamid et al. based on XRD data.^43^ At this stage, the WAXS signal did not show any change (see Figure S3), while the SAXS profile showed a marked change, with an increase in scattered intensity (see Figure 1). A SEM image of the precipitate is shown in Figure S5, showing the formation of a large, aggregated structure with cellulose nanoparticles on the surface. Fitting of the SAXS data gave inconsistent results, which is related to the complexity of the system and also to the fact that the structure of the aggregates is too large to be determined by SAXS. An overview of the changes occurring in the initial steps across the conditions used in this study is reported in Figure S6. As the study of the mechanism of ZIF-8 formation is the main aim of this work, in the following sections we will focus on the final step, where the organic linker is added.

### 3.2 Mechanism of Formation of SCNC/ZIF-8 and ZIF-8 in Water

We followed the time-resolved SAXS and WAXS scattering patterns measured after the addition of the organic linker. An example of data obtained for the synthesis of ZIF-8 in an aqueous solution, in the absence and presence of SCNCs, recorded after the addition of an organic linker, is shown in Figure 2. For a better overview of the experiments used to assess the kinetics, we provide details of the concentrations used in Table S1. We included the time-resolved SAXS and WAXS scattering patterns for all experiments used for data fitting in Figures S7–S14. FITR spectra of the samples prepared using the same conditions as those shown in Figure 2 are included in Figure S15.

**Figure 2:**
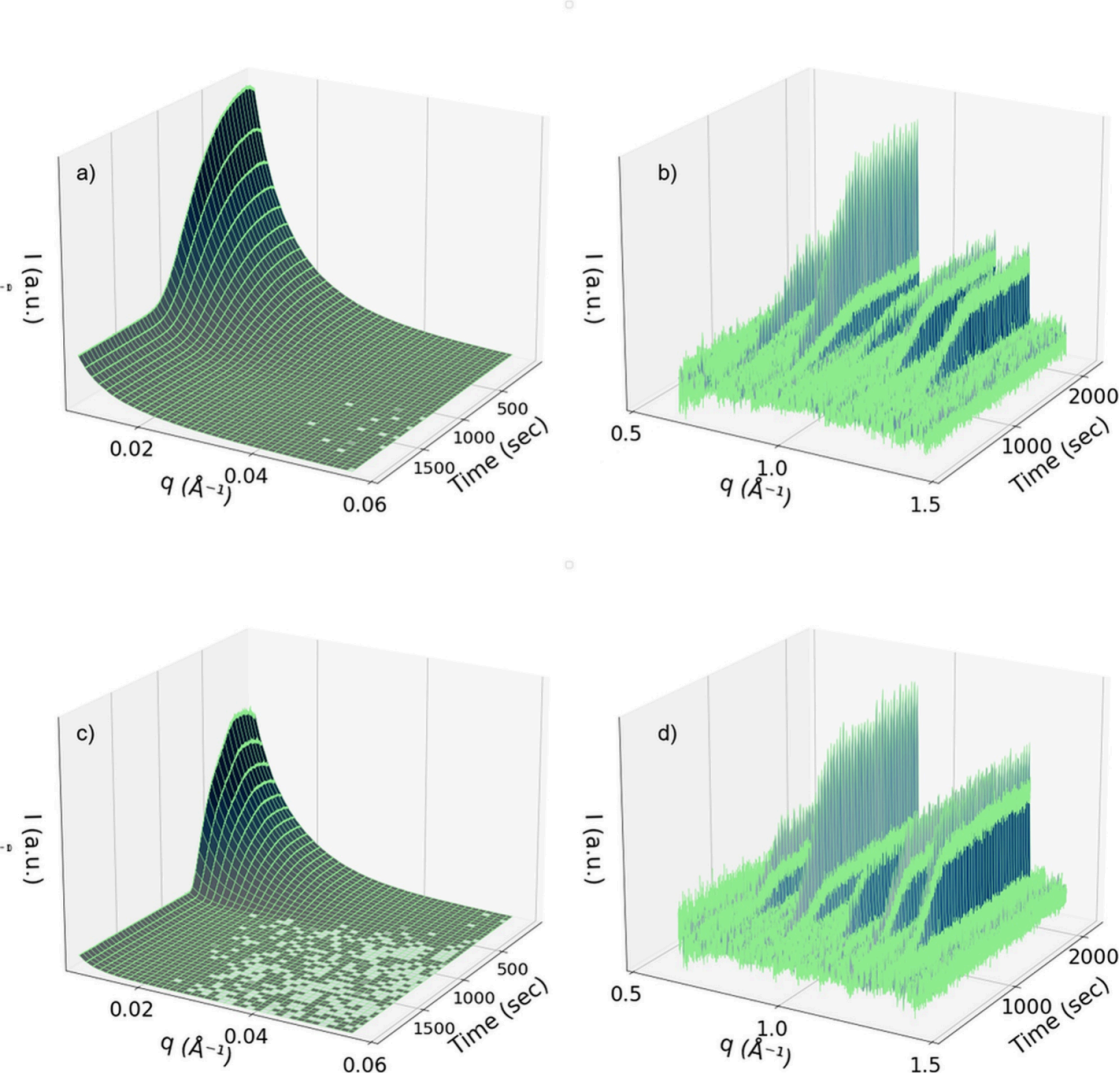
Time-resolved scattering patterns recorded during the formation of ZIF-8 in the presence of SCNCs recorded in the (a) SAXS and (b) WAXS region with 0.2 wt % SCNCs, 9 mM Zn^2+^ and TEA, and 0.6 M Hmim. Scattering patterns recorded during the formation of ZIF-8 in water recorded in the (c) SAXS and (d) WAXS regions. The time interval between succeeding patterns was 0.5 s. The Zn^2+^ and TEA concentration was 14 mM, and the Hmim concentration was 1 M.

It should be mentioned that the linker was injected manually, and we could not follow the reaction during the first minute. This experimental limitation prevents direct observation of the early nucleation steps, which are important for elucidating the mechanism. To better study early stage kinetics, complementary experiments under more diluted conditions were performed, and the results will be presented below. To interpret the WAXS data, we followed the formation and evolution of the ZIF-8 crystalline peaks. More specifically, we followed the evolution of the peak at *q* = 0.93 Å^–1^, corresponding to the (112) plane,^44^ which was the peak with the highest intensity in the recorded region (see Figure S16). The results showing the extent of crystallization as a function of time for the synthesis of both CelloZIF-8 and ZIF-8 are shown in panels a and b, respectively, of Figure 3 and are comparable to previously reported XRD data for the synthesis of ZIF-8.^9,11^

**Figure 3:**
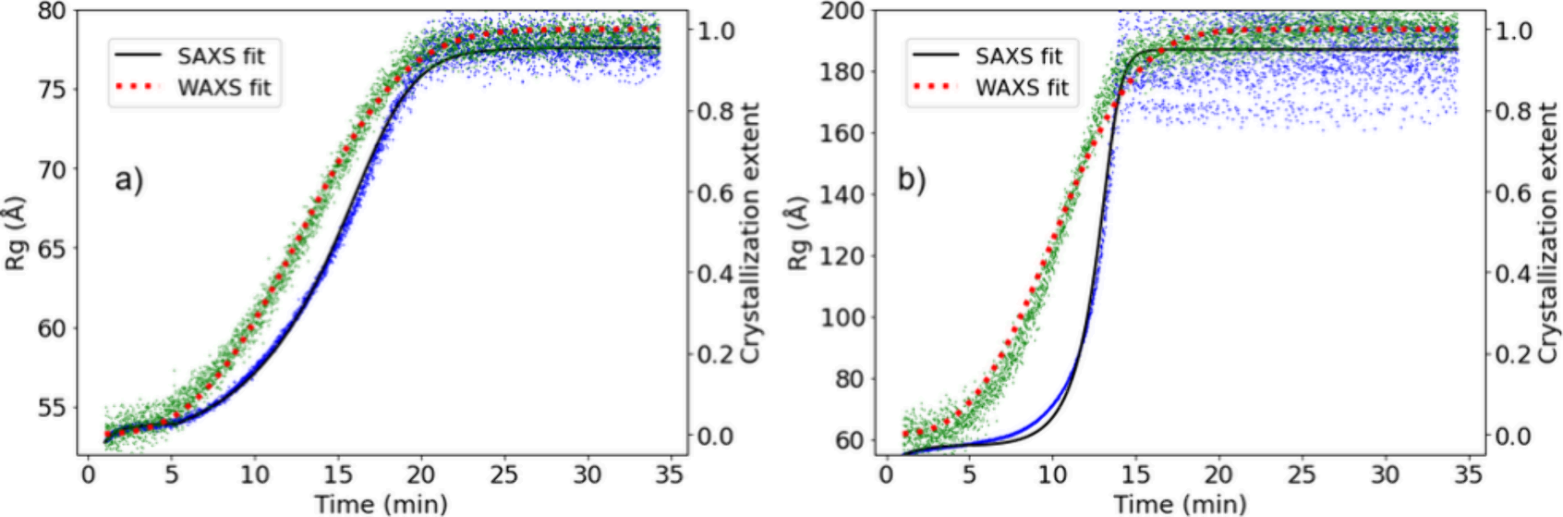
Comparison between the radius of gyration determined from SAXS data (blue squares) and the fit according to a three-step model (solid black line) and crystallization extent determined from WAXS data (green circles) and the fit according to the Avrami model (red dotted line). (a) Synthesis of ZIF-8 in a 0.2 wt % SCNC suspension at 9 mM Zn^2+^ and TEA and 0.6 M Hmim. (b) Synthesis of ZIF-8 in water at 14 mM Zn^2+^ and TEA and 1 M Hmim.

This is the first time that the mechanism of formation of ZIF-8 is studied in the presence of nanocellulose. Past work has focused on the synthesis of pristine ZIF-8 under various experimental conditions, for example, in methanol,^9,10,13,16^ using mechanochemical,^11^ solvothermal,^12^ or sonocrystallization syntheses,^14^ or in water.^20^ Previous works used either the Avrami (AE) model or the Gualtieri model^15^ to fit the data. We used the AE model (see eq 3) to fit WAXS data, considering that both AE and Gualtieri models resulted in a comparable fit. The Avrami constant reflects the overall reaction rate, while the exponent describes the dimensionality and mechanism of crystal growth. The fit of WAXS data according to the AE model is shown in Figure 3. The values of Avrami constants determined in our work were dependent on the concentration of reactants and are reported in Table S2. For the sake of completeness, the analysis was also performed using the (002) crystallographic plane^44^ at *q* = 0.77 Å^–1^, yielding Avrami constants identical to those obtained from the (112) plane (see Table S2). The values of Avrami constants determined in this work were slightly higher than the values reported for the solvothermal reaction at 110 °C,^12^ while the highest value reported in the literature is 0.09 min^–1^, for mechanochemical synthesis of ZIF-8.^11^ In this work, the value of the Avrami exponent was fixed to 3, as it varied between 3 and 4 when set as a fitting parameter, depending on the signal-to-noise ratio, and setting it to 3 did not significantly change the extrapolated values of the Avrami constant. Literature values for the synthesis of ZIF-8 also reported a large variation in the values of the Avrami exponent for the synthesis of ZIF-8, which ranged from 1 to 4.^8^

We compared the WAXS data with the SAXS data. To interpret the SAXS data, the Guiner–Porod model was used (see eqs 4–6), yielding the time evolution of the radius of gyration and the Porod exponent.^35^ *R*_g_ provides a measure of particle size evolution during synthesis, while the Porod exponent reflects the surface roughness and fractal nature of the particles. We chose a shape-independent model due to the complexity of the reaction mixture, and the Guiner–Porod model fitted the experimental data well. Previous work reporting the growth of ZIF-8 in methanol used the same model to interpret SAXS data.^16^ Examples of data fitting are shown in Figure S17. The variation of the radius of gyration as a function of time is shown in Figure 3, showing a sigmoidal-shaped curve for both reactions performed with and without SCNCs. The steeper particle growth observed by SAXS is slightly delayed compared to crystal growth determined by WAXS. This can be explained by considering that, initially, the crystals are small. Then, due to the Ostwald ripening force, smaller crystals dissolve and redeposit on the surface of larger crystals, leading to further growth. The Porod exponent showed a different trend (Figure S18) when the reaction was performed in the presence of nanocellulose, reflecting different structural characteristics. More specifically, the value of the Porod exponent for CelloZIF-8 started from a value of around 3 and decreased to a value closer to 2, which falls into range where mass fractal structures are present. The decrease in the Porod exponent corresponds to the decrease in the fractal dimension. For the synthesis of ZIF-8, the starting and ending values were close to 4, corresponding to smooth surfaces of the scattering particles, and a minimum of around 3 was observed during the synthesis, which corresponds to surface fractals, e.g., rough surface topology.

If we consider the overall kinetics, data obtained by SAXS and WAXS showed a comparable trend and proved the reliability of the Guiner–Porod model. However, a closer look at the initial data points showed a remarkable difference between SAXS and WAXS. For instance, WAXS data did not show any change in the starting part of the curve, since there were not enough units to generate Bragg reflections. Instead, a significant change was observed in the SAXS profile within the first 60 s, showing a decrease in intensity (see Figure 1). In addition, the *R*_g_ values determined in the earlier part of the curve showed an exponential increase (see Figure S19), which could be related to an additional initial step not considered by the Avrami model. In fact, the AE model did not properly fit the SAXS data. An improved fit was given by a model based on the two-step model for particle formation proposed by Finke and Watzky (see eq 7).^36^ This model explains the particle formation considering a slow, continuous nucleation step, characterized by rate constant *k*_1_, followed by an autocatalytic growth phase, governed by *k*_2_.^45^ The Finke–Watzky model has been successfully used in MOF crystallization kinetics, such as MFM300­(Fe), MUF-77, and UMCM-1.^46,47^ A direct comparison is not straightforward due to differences in experimental conditions, but our data showed a comparable range with a lower value of *k*_1_ and a slightly larger value of *k*_2_, reflecting slower nucleation and faster autocatalytic growth. It should be noted that this model did not fit the initial part of our data set. In order to account for this constraint, we introduced an additional initial empirical exponential step, which resulted in an improved three-step fit (see eq 8). A comparison between the models used to fit the SAXS data is included in Figure S19, showing the improvement in fitting accuracy at early times. The values of the kinetic constants and final radius of gyration of particles, determined using two- and three-step models, are reported in Tables S3 and S4.

We characterized the morphology of the CelloZIF-8 particles formed within the first 3 min and at the end of the reaction. SEM images showed the formation of small ZIF-8 nuclei with an average radius of 29 ± 7 nm (see Figure 4a), as well as the fibrous structure of aggregated SCNCs. SEM images of the final CelloZIF-8 are shown in Figure 4b and show particles with a radius of 51 ± 9 nm. Histograms of particle size are shown in panels a and b of Figure S20. The *R*_g_ values determined by SAXS were 5–6 times lower than the *R*_g_ value calculated by SEM. The larger size determined by SEM can be linked to crystal enlargement during drying, which was shown to occur when crystals are not properly separated due to poor mixing or a nonuniform distribution of shear.^48^ We also should consider that the particle radius determined by SAXS may be underestimated due to the complexity of the system, which contains a mixture of different materials scattering at small angles. In addition, the final particles size determined by SAXS is limited by the *q* range (0.04–3 Å^–1^, i.e., a maximum *d* spacing of ∼15.7 nm). Nevertheless, within the limited detection time, initial particle size evaluation is plausible when the particles were still small and fit into the SAXS detection range until the particle size plateau is reached. Furthermore, to determine the kinetics of the reaction, we are taking into account the relative change in *R*_g_, which we consider reliable as it is also supported by the formation of crystallographic peaks in the higher-*q* region and by the trend determined by SEM. In fact, the fitting of data obtained in water without SCNCs resulted in a significantly higher final value of *R*_g_ (see Figure 3), compared to CelloZIF-8. SEM images confirmed this trend, showing that pristine ZIF-8 particles had a radius of 300 ± 100 nm (Figure 5a and histogram in Figure S20). For this system, the difference between the radius of gyration determined by SAXS and SEM is larger and can be related to the fact that the particles decorated with cellulose nanocrystals have a weakened tendency to aggregate due to electrostatic repulsion. To obtain further information about the crystal sizes, we used the Scherrer equation to determine the final crystal size from the WAXS peaks. The data obtained showed a size that is comparable to the SAXS data. The particle size extrapolated from the 112 peak using the Scherrer equation is included in Table S2. Comparable values were obtained by fitting the other peaks. Data do not show a variation in size between ZIF-8 and CelloZIF-8. It should be considered that at high *q* values, the great majority of the scattering is generated by the reactor, which is subtracted and resulted in low-intensity peaks and thus a low signal-to-noise ratio. We also measured DLS of the purified sample (Figure S21), which gave a radius of 200 ± 43 nm, slightly smaller than that determined by SEM. Taken together, Scherrer analysis and DLS confirm that the difference in size is due to crystal aggregation occurring during purification.

**Figure 4:**
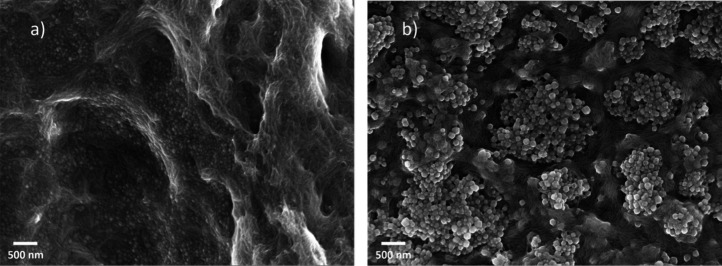
SEM images obtained for the preparation of SCNC/ZIF-8 at 0.2 wt % SCNCs (15 mL), 9 mM Zn^2+^ and TEA, and 0.6 M Hmim. The reaction was stopped after (a) 5 min and (b) 1 h.

**Figure 5:**
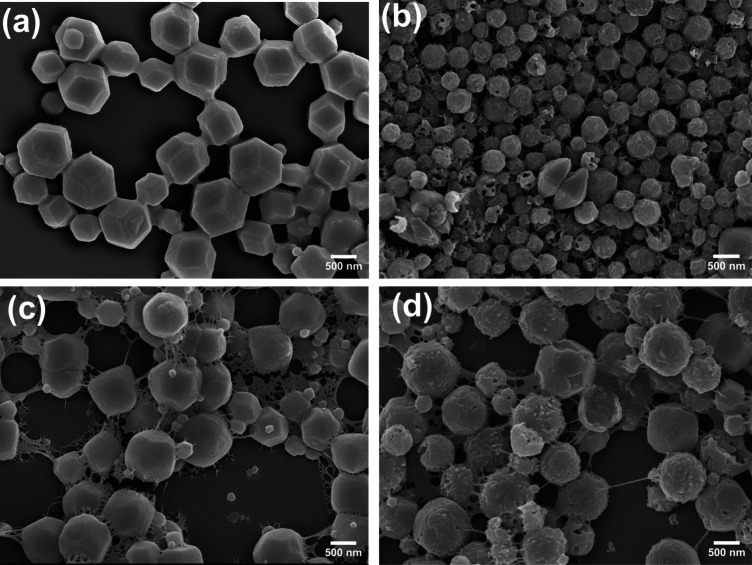
SEM images of (a) pristine ZIF-8 (E8), (b) SCNC/ZIF-8 (E7), (c) TOCNF/ZIF-8 (E5), and (d) PCNF/ZIF-8 (E6). The preparation conditions are listed in Table S1. The reaction time was 1 h. The content of nanocellulose in each hybrid material was 0.02 wt %.

It is difficult to fully understand interactions between ZIF-8 and the nanocellulose support based on SEM, due to the small particle size. However, images of the final CelloZIF-8 product show that the ZIF-8 particles are wrapped on the nanocellulose structure, demonstrating particle–support interactions. The final reaction mixture formed a stable suspension, which provides additional support for the presence of interparticle interactions. However, it should be emphasized that the interparticle interactions shown in SEM images could be driven by solvent removal; thus, more investigations are required to determine interparticle interactions in suspension. The materials were further characterized by gas adsorption, and the results are presented in Table 1 and Figure S22.

**Table 1:** Surface Areas, Porosities, and Morphological Data of ZIF-8 and Hybrid Nanocellulose/ZIF-8 Materials^a^

name	ZIF-8/NC mass fraction	NC type	surface area^a^ (m^2^/g)	micropore volume^b^ (cm^3^/g)	external surface^b^ (m^2^/g)	particle radius^c^ (nm)
E8	100	–	1576	0.55	32	330 ± 100
E1	1.3	SCNC	1197	0.41	51	51 ± 9
E3	2.6	SCNC	1182	0.41	52	120 ± 30
E7	25.7	SCNC	1520	0.53	35	160 ± 40
E6	25.7	PCNF	1537	0.53	42	360 ± 120
E5	25.7	TOCNF	1550	0.53	40	260 ± 120

a The mass of the hybrid material was used to determine the surface area, micropore volume, and external surface. Details of the experimental conditions of each sample are reported in Table S1 according to the name.

b Determined with the BET model employing Rouquerol criteria.

c Calculated by employing the t-plot model.

d Average size of 86–120 particles determined from SEM images.

The isotherm, surface area, and size of the main pore of the pristine ZIF-8 material match well with the results published previously.^49^ It is a typical microporous material type I isotherm with a two-step feature in the micropore regime that was explained as a reorganization of the adsorbed gas molecules at a certain threshold pressure.^49^ Pristine ZIF-8 has a slightly larger surface area and micropore volume than most of the CelloZIF-8 materials, as shown in Table 1 (see Figure S22 for isotherms and pore size distribution graphs). The decrease in both parameters for CelloZIF-8 was associated with the presence of nanocellulose. The decrease is more significant at a low ZIF-8/cellulose ratio, but the hybrids can be considered as dominantly microporous (see Figure S22), large-surface area materials. Data do not show a clear correlation with the particle size because the surface of the internal micropores in ZIF-8 is the main contributor to the surface area, and that will change only slightly with particle size. The external surface of the hybrid materials is larger than that for pristine ZIF-8, but at these relatively large particle sizes, this increase in the external surface will not make a significant contribution to the whole surface area. The isotherms of the hybrid materials show the presence of larger pores (large mesopores/macropores) that is confirmed by the pore size distribution in the BJH model (see Figure S22). These pores can be related to interparticle voids between cellulose fibers and ZIF-8 particles or between agglomerated ZIF-8 particles, and the large pores could also be introduced by the nanocellulose.

To better understand the process occurring in the initial part of the reaction, we used a custom-built syringe pump to inject the linker and follow the initial changes. As the volume was quite large to be injected in less than 60 s without excessively increasing the pressure in the syringe, we performed a slow injection. We used a smaller amount of both Zn­(NO_3_)_2_ and Hmim to avoid the possibility that the growth would start before the addition of all of the linkers. The results showing the evolution of *R*_g_ as a function of time under these conditions are shown in Figure 6b, which show the specific time when the organic linker is added, and SAXS data are shown in Figures S23 and S24. After the injection of all of the linkers, we further followed the reaction for 1 h, but we did not obtain the final growth phase under these conditions. As soon as the organic linker was added, the zinc oxide precipitate dissolved, leading to a decrease in *R*_g_, and the formation of elementary particles (molecular Zn­(Him)_2_) occurs simultaneously. After 10–15 s, the *R*_g_ value reached a minimum, and we associate this step with the aggregation of elementary particles into amorphous clusters. Then, we measured a rapid increase in *R*_g_, which can be associated with the crystallization of primary clusters to form nuclei. It should be considered that at this stage both the formation of clusters and their evolution into nuclei occur simultaneously, as we are slowly adding organic linkers. The maximum inflection point for *R*_g_ evolution in Figure 6b occurred when the Hmim/Zn^2+^ molar ratio was 2.5, close to the stoichiometric number for molecular ZIF-8, indicating that molecular ZIF-8 is formed as soon as the organic linker is added, and the molecules quickly aggregate forming primary clusters. After that point, the organic linker is in excess, and we can assume that at this stage only the nucleation affects the changes in the SAXS scattering profile. Here, a small change in the scattering pattern and a small increase in *R*_g_ were determined. We expect that within this stage the ZIF-8 nuclei slowly grow, possibly through Ostwald ripening, which was previously reported for ZIF-8 formation.^8^ Although these nuclei look amorphous due to the low resolution of WAXS in solution, it was proposed by Venna et al., who studied the mechanism of formation of ZIF-8 in methanol, that such particles might have low crystallinity.^9^ Comparable evidence was reported for the synthesis ZIF-71.^29^ The crystallinity of the ZIF-8 nuclei was confirmed by Liu et al., who proposed a three-step mechanism for ZIF-8 nucleation, starting with a phase separation of the ZIF-8 molecules into solute-rich regions followed by condensation into amorphous primary particles, which would then crystallize to form ZIF-8 nuclei. We performed powder XRD of the purified samples to confirm the low crystallinity of the nuclei formed in the initial time of the reaction (Figure S25). Overall, our proposed mechanism is in line with the mechanism of nucleation proposed by Liu et al.,^20^ even though we were not able to use our data to differentiate between phase separation and condensation, as both steps occurred too fast, and both steps were included in the initial empirical exponential step proposed in our model. A schematic representation of our proposed mechanism supported by the experimental data is shown in Figure 6. We associate the three steps in the experiments performed in Figure 6b (first turning point) and Figure 6c, which show a selected part of the SAXS data shown in Figure 3a.

**Figure 6:**
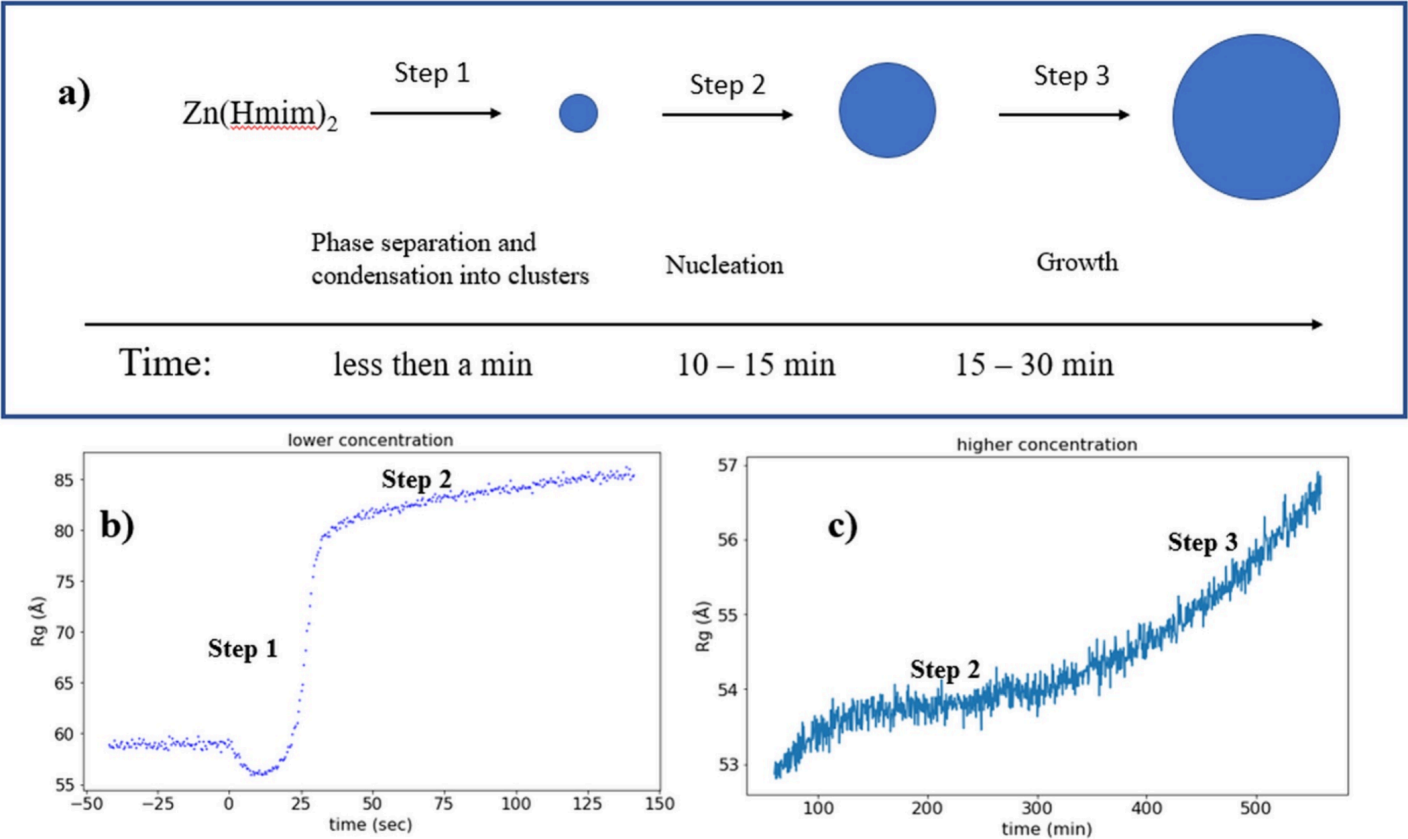
(a) Proposed scheme for the mechanism of formation of ZIF-8 in water in the absence and presence of nanocellulose. Relation between the proposed mechanism of formation and our experimental data obtained at (b) a lower linker concentration with 15 mL of 0.2% SCNC, 0.2 mL of 0.84 M Zn­(NO_3_)_2_, 0.025 mL of TEA, and 1 mL of 3 M Hmim and (c) a higher linker concentration with 15 mL 0.2% SCNC, 0.2 mL of 0.84 M Zn­(NO_3_)_2_, 0.025 mL of TEA, and 4 mL of 3 M Hmim. The flow rate in panel b was 12.8 mL h^–1^.

### 3.3 Effect of the Concentration of Reagents

The kinetics of formation of CelloZIF-8 was determined by varying the starting concentrations of the reagents. Data obtained upon variation of the concentration of the reagents are shown in panels a and d of Figure 7. Our data show that the rate of reaction for the formation of ZIF-8 in water is dependent on the concentration of the reagents. In fact, the value of the Avrami constant extrapolated from the WAXS data increased as a function of the starting concentration of both the precursor and the linker (see Table S2). The two- and three-step models, which take into account the effect of the concentration of the precursor on *k*_2_ and *k*_3_, respectively, also resulted in an increase in the kinetic constants as a function of the reactant concentrations (see Tables S3 and S4), taking into account the autocatalytic growth constants (*k*_2_ in the two-step model and *k*_3_ in the three-step model) and the initial empirical step in the three-step model. Our findings are in line with the results presented by Saha et al., who studied the effect of the linker-to-metal concentration ratio in the kinetics of formation of ZIF-71 in propanol, showing that the rate of the reaction increased with the linker-to-metal concentration ratio.^29^ The values of the nucleation rate constants (*k*_1_ in the two-step model and *k*_2_ in the three-step model) were very small and exhibited significant variation depending on the experimental conditions used. In addition, experiments performed at lower concentrations, such as the experiment shown in Figure 6b, did not reach the growth phase in the recorded time (around 1 h). The final values of *R*_g_ did not show a large variation as a function of the metal precursor concentration under the studied conditions. Even if our data are sufficient to highlight a clear trend that shows an increase in the rate of reaction as a function of the concentration of starting reagents, we could not fully understand the effect of the concentration of reactants or extrapolate reaction orders. For that, a larger number of data is required, varying the concentration of the various reagents, which was not possible due to the limited available time at the beamline.

**Figure 7:**
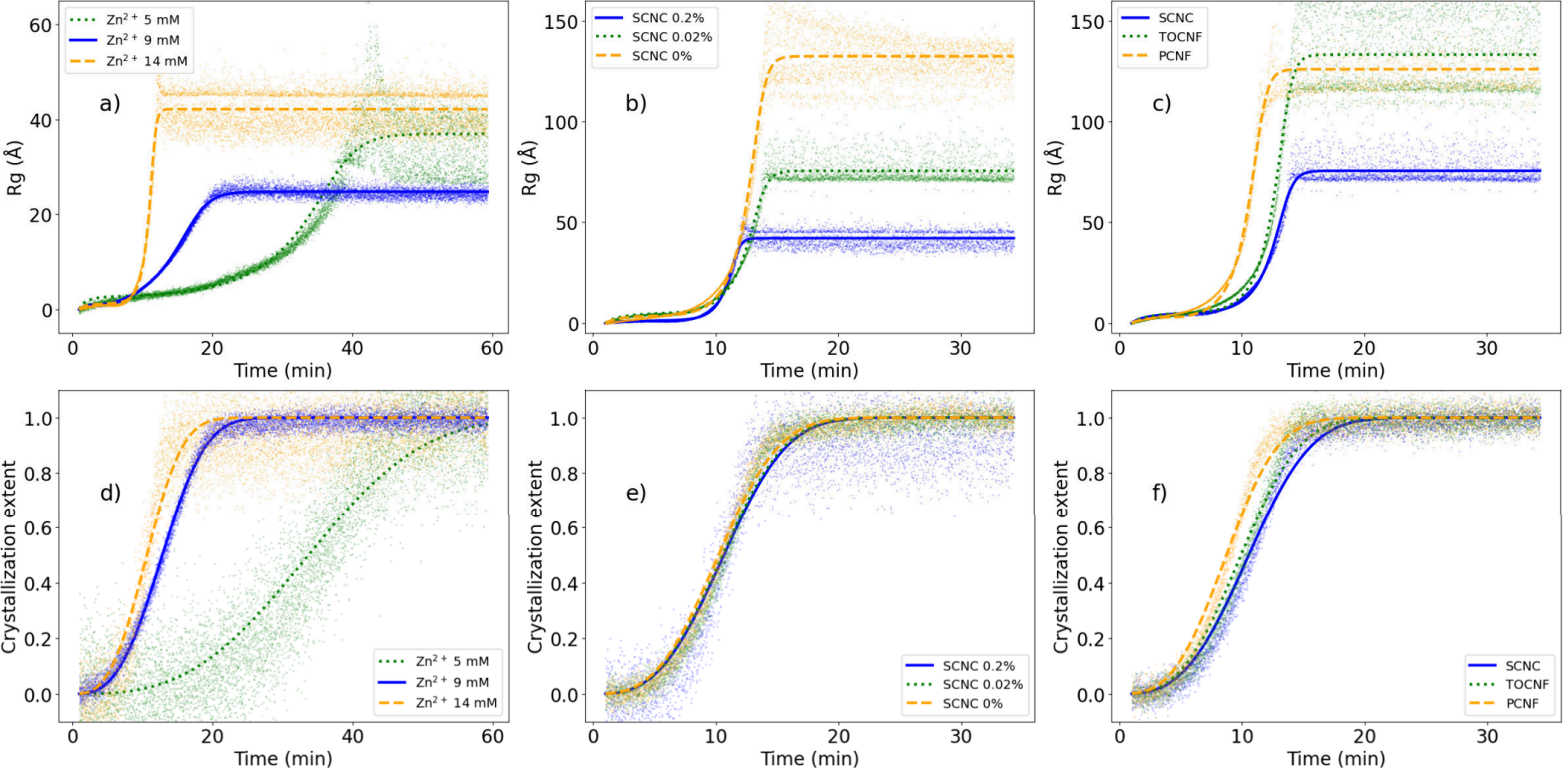
Time evolution of the (a–c) radius of gyration and (d–f) crystallization extent. Time evolution of CelloZIF-8 synthesis as a function of the starting concentration of the precursor: E1 (blue solid line), E2 (green dotted line), and E3 (orange dashed line). Time evolution of CelloZIF-8 synthesis as a function of CNC concentration: E3 (blue solid line), E7 (green dotted line), and E8 (orange dashed line). Time evolution of CelloZIF-8 synthesis as a function of nanocellulose functionality: E7 (blue solid line), E5 (green dotted line), and E6 (orange dashed line). Synthesis conditions according to the experiment names (E1–E8) are reported in Table S1.

### 3.4 Effect of SCNC Concentration

We also studied the effect of the SCNC concentration (see Figure 7b,e). The results showed little or no variation in the reaction rates when the concentration of reactants was the same, suggesting that SCNCs do not kinetically control ZIF-8 formation under these dilute conditions (≤0.2 wt %). The choice of working concentrations was guided by experimental constraints to maintain near-Newtonian conditions, essential to ensure proper stirring and avoid beam damage. None of the samples looked viscous under the conditions used. As support, literature on sulfated CNCs confirms that dilute regimes (<0.5 wt %) exhibit Newtonian behavior and present high colloidal stability under moderate ionic strengths (>20 mM).^50^

The *R*_g_ values determined by SAXS showed variation of the final value of the radius of gyration, which decreased with an increase in SCNC concentration, highlighting their role in interfacial assembly rather than reaction kinetics. It should be considered that the data presented are valid under the dilute conditions, while at higher concentrations, other factors such as viscosity and shear effects could alter the reaction mechanism. The synthesis performed at 0.02% SCNC gave particles of 160 ± 40 nm by SEM (see Figure 5b and Figure S20), which is in line with the trend of *R*_g_ versus time determined by SAXS. We expect that the interactions between SCNC and ZIF-8 are responsible for the smaller size of ZIF-8 obtained. It is possible that the amphiphilic character of SCNCs is responsible for binding on the solid–liquid ZIF-8–water interface, forming a network structure that acts as a mechanical barrier against precipitation and reducing aggregation. A similar mechanism was reported for stabilization of an oil-in-water emulsion by CNCs.^51^ In addition, the trend for the Porod exponent also showed a remarkable change when the synthesis of ZIF-8 was performed without SCNCs (see Figure S26a), which further highlights the structural differences in the system. Interestingly, it is possible to observe cellulose nanocrystals attached on the surface of the ZIF-8 crystals from the SEM image shown in Figure 5b, even at 0.02 wt % SCNC, confirming the presence of interparticle interactions upon drying. In addition, the data presented in Table 1 confirm that the porosity and surface area are unaffected when the concentration of SCNC was 0.02 wt %, showing the high potential of CelloZIF-8 for application where a large surface area and high porosity are required, such as gas and water adsorption.

### 3.5 Effect of Nanocellulose Shape and Functionality

We also studied the effect of using larger cellulose nanofibers and varying surface functionalities. Previous work from our group has reported the experimental procedure for the preparation of CelloZIF-8 in the presence of TOCNFs.^3^ In this work, we used both TOCNFs and PCNFs. To perform the scattering experiments with both cellulose nanofibers, we had to decrease the concentration of nanocellulose by 10 times since at a concentration of 0.2% the TOCNF/PCNF would form a gel phase, preventing adequate mixing, which is crucial to avoid radiation damage within the experimental time. In this work, we used TOCNF/PCNF at 0.02 wt % bearing either carboxylic or phosphate functionalities. The results are shown in Figure 7c–f. For both TOCNFs and PCNFs, the kinetics was comparable to that of the formation of pristine ZIF-8, showing that the presence of nanocellulose did not contribute significantly to the rate of reaction. The value of *R*_g_ determined was also in the same range as that of pristine ZIF-8, while the final *R*_g_ determined for SCNC was lower. Also, the time evolution of the Porod exponent determined for both TOCNFs and PCNFs showed a trend that was comparable to that of pristine ZIF-8 (see Figure S26b). For CelloZIF-8 obtained at 0.02% SCNC, the variation of the Porod exponent was comparable to that of CelloZIF-8 obtained at 0.2% SCNC, and it is possible that this is related to the larger amount of charged surface groups in both PCNFs and TOCNFs at the pH used (which was between 8 and 9), compared to the case for sulfated SCNCs. It is possible that the higher hydrophilic character of the highly charged CNFs resulted in weaker interactions with the hydrophobic ZIF-8 and is responsible for the larger size of the TOCNF/ZIF-8 and PCNF/ZIF-8 particles, compared to SCNCs. More specifically, the sizes in terms of the radius of the TOCNF/ZIF-8 and PCNF/ZIF-8 hybrid materials determined from SEM (see panels c and d of Figure 5 and histograms in Figure S20) were 260 ± 100 and 350 ± 100 nm, respectively, in the same range of pristine ZIF-8. As shown in Table 1, values of both surface areas and pore volumes are in line with pristine ZIF-8, showing that under this condition we can form hybrid materials retaining both a large surface area and high porosity of pristine ZIF-8. SEM images of both TOCNF/ZIF-8 and PCNF/ZIF-8 presented in panels c and d, respectively, of Figure 5 show that the cellulose nanofibers are present on the surface of the nanocrystals under drying conditions. In particular, PCNF fibers fully encapsulate the ZIF-8 particles under the conditions used. Also, the network structure formed by the nanofibers interconnecting the CelloZIF-8 particles is fully visible. However, more work is required to fully understand the effect of NC functional groups and size upon interaction with ZIF-8 in suspension and to elucidate the effect of interparticle interaction on the final particle size.

## 4 Conclusions

We successfully used for the first time a combination of in operando SAXS and WAXS to study the synthesis of ZIF-8 in water, in the absence and presence of nanocellulose, and we reported the effect of nanocellulose surface functionality, morphology, and reagent concentration. Our approach was highly innovative, as we succeeded in obtaining reproducible measurements, even in the challenging environment of a dense nanocellulose matrix, which produces strong scattering at small angles. By combining SAXS and WAXS, we validated particle growth trends across a wide range of sizes, pushing the capabilities of these techniques beyond their usual limitations. We observed an initial increase in the form factor after Zn^2+^ addition, followed by a strong increase in low-*q* scattering upon TEA addition, suggesting the formation of an amorphous precursor. By comparing the time evolution of the radius of gyration from SAXS and the crystalline peaks from WAXS, we resolved the kinetics of ZIF-8 crystallization in both systems. Based on the SAXS data, a model for CelloZIF-8 formation in water was proposed, where the fast formation of primary nanoparticles is followed by the development of medium-sized aggregates before their evolution into the final crystalline product. SEM imaging supported this mechanism, showing that small particles present at early reaction times progressively merged into larger structures. We found that the concentration of the metal precursor and linker significantly influenced the crystallization kinetics, while the presence of nanocellulose had a negligible impact on the reaction rate but had a strong influence on the particle size of the final ZIF-8 crystals. Higher nanocellulose concentrations led to smaller ZIF-8 crystals, an effect associated with interparticle interactions that could be viewed by SEM after drying. Further work is required to determine interparticle interactions in the solvent media. Importantly, the hybrid CelloZIF-8 material maintained a large surface area and a high porosity at lower NC concentrations (0.02 wt %), though a minor decrease was observed at higher concentrations (0.2 wt % SCNC). We also found that different nanocellulose types, including longer, highly charged TOCNF and PCNF cellulose nanofibers, did not significantly alter the kinetics of ZIF-8 formation at low concentrations. However, they effectively coated the final ZIF-8 crystals without impacting the overall size or porosity, demonstrating the possibility of engineering hybrid materials that preserve the advantageous features of ZIF-8 while adding new functionality through the cellulose network. These results highlight that SAXS/WAXS, when carefully applied, can successfully unravel nucleation and growth mechanisms even in complex, highly scattering environments, opening a new path for the study of hybrid material formation under realistic conditions. Our findings advance the understanding of MOF synthesis in aqueous and biobased media, providing a novel mechanism of formation. In the future, deeper investigations are needed to characterize the potential impact on the mechanical properties, stability, and performance in specific applications. Open questions remain about the interparticle interactions in solution, the behavior of these hybrids at higher nanocellulose loadings, the role of different surface functionalities, and the extent to which the cellulose network can be used to direct or template MOF crystal growth more actively without a major loss of porosity. The effect of functionality will be the subject of our future investigation from a force spectroscopy point of view.

## Supplementary Material



## Notes

Image of the reactor; AFM images of the nanocellulose suspension; preliminary WAXS pattern; SAXS data for the addition of Zn; SEM image of the solid material formed after the addition of TEA to SCNC-Zn; Preliminary SAXS pattern; 3D time-resolved scattering patterns recorded via SAXS/WAXS for all conditions used; FTIR spectra of SCNC/ZIF-8 and ZIF-8; WAXS scattering pattern at selected times for the synthesis of CelloZIF-8 and ZIF-8; SAXS scattering pattern and fitting at selected times for all conditions used; variation of the Porod exponent during the synthesis of CelloZIF-8 and ZIF-8; comparison of models used for fitting; histograms showing the particle size distributions; dynamic light scattering profile and log-normal distribution of ZIF-8; adsorption isotherms and pore size distributions; examples of SAXS scattering patterns, 3D time-resolved scattering patterns, and selected Guiner–Porod fits for data not used for modeling; powder XRD of ZIF-8; time evolution of the Porod exponent as a function of SCNC concentration and surface functionality; and tables reporting experimental conditions and fitting data from the Avrami, two-step, and three-step models (PDF)

## ∇

Departament d’Enginyeria Química, Agrària i Tecnologia Agroalimentària, University of Girona, C/Maria Aurèlia Capmany, 61 Campus Montilivi, 17003 Girona, Spain

## References

[1] Abdelhamid H. N., Sultan S., Mathew A. P. (2023). Three-Dimensional
Printing of Cellulose/Covalent
Organic Frameworks (CelloCOFs) for CO2 Adsorption and Water Treatment. ACS Appl. Mater. Interfaces.

[2] Gong X., Zhang L., Liu Y., Zhu M. (2023). A Review on Zeolitic
Imidazolate Framework-8 Based Materials with Special Wettability for
Oil/Water Separation. J. Environ. Chem. Eng..

[3] Abdelhamid H. N., Georgouvelas D., Edlund U., Mathew A. P. (2022). CelloZIFPaper: Cellulose-ZIF
Hybrid Paper for Heavy Metal Removal and Electrochemical Sensing. Chem. Eng. J..

[4] Xue W., Zhou Q., Li F., Ondon B. S. (2019). Zeolitic Imidazolate
Framework-8 (ZIF-8) as Robust Catalyst for Oxygen Reduction Reaction
in Microbial Fuel Cells. J. Power Sources.

[5] Deacon A., Briquet L., Malankowska M., Massingberd-Mundy F., Rudić S., Hyde T. I., Cavaye H., Coronas J., Poulston S., Johnson T. (2022). Understanding the ZIF-L
to ZIF-8
Transformation from Fundamentals to Fully Costed Kilogram-Scale Production. Commun. Chem..

[6] Kida K., Okita M., Fujita K., Tanaka S., Miyake Y. (2013). Formation
of High Crystalline ZIF-8 in an Aqueous Solution. CrystEngComm.

[7] Lee Y. R., Jang M. S., Cho H. Y., Kwon H. J., Kim S., Ahn W. S. (2015). ZIF-8: A Comparison of Synthesis Methods. Chemical Engineering Journal.

[8] Van
Vleet M. J., Weng T., Li X., Schmidt J. R. (2018). In Situ,
Time-Resolved, and Mechanistic Studies of Metal-Organic Framework
Nucleation and Growth. Chem. Rev..

[9] Venna S. R., Jasinski J. B., Carreon M. A. (2010). Structural Evolution of Zeolitic
Imidazolate Framework-8. J. Am. Chem. Soc..

[10] Zhu M., Venna S. R., Jasinski J. B., Carreon M. A. (2011). Room-Temperature
Synthesis of ZIF-8: The Coexistence of ZnO Nanoneedles. Chem. Mater..

[11] Friščić T., Halasz I., Beldon P. J., Belenguer A. M., Adams F., Kimber S. A. J., Honkimäki V., Dinnebier R. E. (2013). Real-Time and in Situ Monitoring of Mechanochemical
Milling Reactions. Nat. Chem..

[12] Moh P. Y., Brenda M., Anderson M. W., Attfield M. P. (2013). Crystallisation
of Solvothermally Synthesised ZIF-8 Investigated at the Bulk, Single
Crystal and Surface Level. CrystEngComm.

[13] Cravillon J., Schröder C. A., Bux H., Rothkirch A., Caro J., Wiebcke M. (2012). Formate Modulated Solvothermal
Synthesis
of ZIF-8 Investigated Using Time-Resolved in Situ X-Ray Diffraction
and Scanning Electron Microscopy. CrystEngComm.

[14] Seoane B., Zamaro J. M., Tellez C., Coronas J. (2012). Sonocrystallization
of Zeolitic Imidazolate Frameworks (ZIF-7, ZIF-8, ZIF-11 and ZIF-20). CrystEngComm.

[15] Gualtieri A. F. (2001). Synthesis
of Sodium Zeolites from a Natural Halloysite. Phys. Chem. Min.

[16] Cravillon J., Schröder C. A., Nayuk R., Gummel J., Huber K., Wiebcke M. (2011). Fast Nucleation
and Growth of ZIF-8 Nanocrystals Monitored
by Time-Resolved in Situ Small-Angle and Wide-Angle X-Ray Scattering. Angewandte Chemie - International Edition.

[17] Moh P. Y., Cubillas P., Anderson M. W., Attfield M. P. (2011). Revelation of the
Molecular Assembly of the Nanoporous Metal Organic Framework ZIF-8. J. Am. Chem. Soc..

[18] Allegretto J. A., Onna D., Bilmes S. A., Azzaroni O., Rafti M. (2024). Unified Roadmap
for ZIF-8 Nucleation and Growth: Machine Learning Analysis of Synthetic
Variables and Their Impact on Particle Size and Morphology. Chem. Mater..

[19] Pan Y., Liu Y., Zeng G., Zhao L., Lai Z. (2011). Rapid Synthesis of
Zeolitic Imidazolate Framework-8 (ZIF-8) Nanocrystals in an Aqueous
System. Chem. Commun..

[20] Liu X., Chee S. W., Raj S., Sawczyk M., Král P., Mirsaidov U. (2021). Three-Step
Nucleation of Metal-Organic Framework Nanocrystals. Proc. Natl. Acad. Sci. U. S. A..

[21] Sultan S., Abdelhamid H. N., Zou X., Mathew A. P. (2019). CelloMOF: Nanocellulose
Enabled 3D Printing of Metal-Organic Frameworks. Adv. Funct. Mater..

[22] Dai H., Yuan X., Jiang L., Wang H., Zhang J., Zhang J., Xiong T. (2021). Recent Advances
on ZIF-8 Composites
for Adsorption and Photocatalytic Wastewater Pollutant Removal: Fabrication,
Applications and Perspective. Coord. Chem. Rev..

[23] Abdelhamid H. N., Mathew A. P. (2022). Cellulose-Metal
Organic Frameworks (CelloMOFs) Hybrid
Materials and Their Multifaceted Applications: A Review. Coord. Chem. Rev..

[24] Ma X., Lou Y., Chen X. B., Shi Z., Xu Y. (2019). Multifunctional Flexible
Composite Aerogels Constructed through In-Situ Growth of Metal-Organic
Framework Nanoparticles on Bacterial Cellulose. Chemical Engineering Journal.

[25] Tan C., Lee M. C., Arshadi M., Azizi M., Abbaspourrad A. (2020). A Spiderweb-Like
Metal-Organic Framework Multifunctional Foam. Angewandte Chemie - International Edition.

[26] Nasser
Abdelhamid H., Sultan S., Mathew A. P. (2023). Binder-Free Three-Dimensional
(3D) Printing of Cellulose-ZIF8 (CelloZIF-8) for Water Treatment and
Carbon Dioxide (CO_2_) Adsorption. Chem. Eng. J..

[27] Lu Y., Liu C., Mei C., Sun J., Lee J., Wu Q., Hubbe M. A., Li M.-C. (2022). Recent
Advances in Metal Organic
Framework and Cellulose Nanomaterial Composites. Coord. Chem. Rev..

[28] Goesten M. G., Stavitski E., Juan-Alcañiz J., Martiñez-Joaristi A., Petukhov A. V., Kapteijn F., Gascon J. (2013). Small-Angle X-Ray Scattering
Documents the Growth of Metal-Organic Frameworks. Catal. Today.

[29] Saha S., Springer S., Schweinefuß M. E., Pontoni D., Wiebcke M., Huber K. (2016). Insight into Fast Nucleation
and Growth of Zeolitic Imidazolate Framework-71
by in Situ Time-Resolved Light and X-Ray Scattering Experiments. Cryst. Growth Des.

[30] Khalili H., Monti S., Pesquet E., Jaworski A., Lombardo S., Mathew A. P. (2024). Nanocellulose-Bovine
Serum Albumin Interactions in
an Aqueous Medium: Investigations Using In Situ Nanocolloidal Probe
Microscopy and Reactive Molecular Dynamics Simulations. Biomacromolecules.

[31] Nygård K., McDonald S. A., González J. B., Haghighat V., Appel C., Larsson E., Ghanbari R., Viljanen M., Silva J., Malki S., Li Y., Silva V., Weninger C., Engelmann F., Jeppsson T., Felcsuti G., Rosén T., Gordeyeva K., Söderberg L. D., Dierks H., Zhang Y., Yao Z., Yang R., Asimakopoulou E. M., Rogalinski J. K., Wallentin J., Villanueva-Perez P., Krüger R., Dreier T., Bech M., Liebi M., Bek M., Kádár R., Terry A. E., Tarawneh H., Ilinski P., Malmqvist J., Cerenius Y. (2024). ForMAX - a Beamline for Multiscale
and Multimodal Structural
Characterization of Hierarchical Materials. J. Synchrotron Radiat.

[32] Avrami M. (1939). Kinetics of
Phase Change. I General Theory. J. Chem. Phys..

[33] Nayuk R., Huber K. (2012). Formfactors of Hollow
and Massive Rectangular Parallelepipeds at
Variable Degree of Anisometry. Z. Phys. Chem..

[34] Lombardo S., Eyley S., Schütz C., van Gorp H., Rosenfeldt S., Van den Mooter G., Thielemans W. (2017). Thermodynamic Study of the Interaction
of Bovine Serum Albumin and Amino Acids with Cellulose Nanocrystals. Langmuir.

[35] Hammouda B. (2010). A New Guinier-Porod
Model. J. Appl. Crystallogr..

[36] Whitehead C. B., Özkar S., Finke R. G. (2019). LaMer’s 1950 Model for Particle
Formation of Instantaneous Nucleation and Diffusion-Controlled Growth:
A Historical Look at the Model’s Origins, Assumptions, Equations,
and Underlying Sulfur Sol Formation Kinetics Data. Chem. Mater..

[37] Schütz C., Van Rie J., Eyley S., Gençer A., van Gorp H., Rosenfeldt S., Kang K., Thielemans W. (2018). Effect of
Source on the Properties and Behavior of Cellulose Nanocrystal Suspensions. ACS Sustainable Chem. Eng..

[38] Paul H. R., Bera M. K., Macke N., Rowan S. J., Tirrell M. V. (2024). Quantitative
Determination of Metal Ion Adsorption on Cellulose Nanocrystals Surfaces. ACS Nano.

[39] Valencia L., Kumar S., Nomena E. M., Salazar-Alvarez G., Mathew A. P. (2020). In-Situ Growth of Metal Oxide Nanoparticles
on Cellulose
Nanofibrils for Dye Removal and Antimicrobial Applications. ACS Appl. Nano Mater..

[40] Valencia L., Nomena E. M., Monti S., Rosas-Arbelaez W., Mathew A. P., Kumar S., Velikov K. P. (2020). Multivalent
Ion-Induced
Re-Entrant Transition of Carboxylated Cellulose Nanofibrils and Its
Influence on Nanomaterials’ Properties. Nanoscale.

[41] Lombardo S., Gençer A., Schütz C., Van Rie J., Eyley S., Thielemans W. (2019). Thermodynamic Study of Ion-Driven Aggregation of Cellulose
Nanocrystals. Biomacromolecules.

[42] Grachev V., Lombardo S., Bartic C., Thielemans W. (2024). Thermodynamics
of Interactions between Cellulose Nanocrystals and Monovalent Counterions. Carbohydr. Polym..

[43] Abdelhamid H. N., Huang Z., El-Zohry A. M., Zheng H., Zou X. (2017). A Fast and
Scalable Approach for Synthesis of Hierarchical Porous Zeolitic Imidazolate
Frameworks and One-Pot Encapsulation of Target Molecules. Inorg. Chem..

[44] Kaur H., Mohanta G. C., Gupta V., Kukkar D., Tyagi S. (2017). Synthesis
and Characterization of ZIF-8 Nanoparticles for Controlled Release
of 6-Mercaptopurine Drug. J. Drug Deliv Sci.
Technol..

[45] Watzky M. A., Finke R. G. (1997). Transition Metal Nanocluster Formation Kinetic and
Mechanistic Studies. A New Mechanism When Hydrogen Is the Reductant:
Slow, Continuous Nucleation and Fast Autocatalytic Surface Growth. J. Am. Chem. Soc..

[46] He B., Macreadie L. K., Gardiner J., Telfer S. G., Hill M. R. (2021). In Situ
Investigation of Multicomponent MOF Crystallization during Rapid Continuous
Flow Synthesis. ACS Appl. Mater. Interfaces.

[47] Godfrey H. G. W., Briggs L., Han X., Trenholme W. J. F., Morris C. G., Savage M., Kimberley L., Magdysyuk O. V., Drakopoulos M., Murray C. A., Tang C. C., Frogley M. D., Cinque G., Yang S., Schröder M. (2019). Analysis by
Synchrotron X-Ray Scattering of the Kinetics of Formation of an Fe-Based
Metal-Organic Framework with High CO_2_ Adsorption. APL Mater..

[48] Lekhal A., Girard K. P., Brown M. A., Kiang S., Glasser B. J., Khinast J. G. (2003). Impact of Agitated
Drying on Crystal Morphology: KCl-Water
System. Powder Technol..

[49] Park K. S., Ni Z., Côté A. P., Choi J. Y., Huang R., Uribe-Romo F. J., Chae H. K., O’keeffe M., Yaghi O. M. (2006). Exceptional Chemical
and Thermal Stability of Zeolitic
Imidazolate Frameworks. Proc. Natl. Acad. Sci.
U. S. A..

[50] Araki J., Wada M., Kuga S., Okano T. (1998). Flow Properties of
Microcrystalline Cellulose Suspension Prepared by Acid Treatment of
Native Cellulose. Colloids Surf. A Physicochem
Eng. Asp.

[51] Lombardo S., Villares A. (2020). Engineered Multilayer Microcapsules
Based on Polysaccharides
Nanomaterials. Molecules.

