# Supplementary material for: In Situ Formation of Zeolitic Imidazolate Frameworks on Nanocellulose Revealed by Time-Resolved Synchrotron Small-Angle and Wide-Angle X‑ray Scattering
Source: ACS Appl Mater Interfaces. 2025 Aug 18;17(34):48976–88. doi: 10.1021/acsami.5c10734 (PMC12400270; doi:10.1021/acsami.5c10734)
Supplement: Supplementary file 1 [file am5c10734_si_001.pdf]

Supporting information for:

In-situ Formation of Zeolitic Imidazolate Frameworks on Nanocellulose Revealed by Time-resolved Synchrotron Small-angle and Wide-angle X-ray Scattering

Salvatore Lombardo,*[a] Houssine Khalili,[a] Shun Yu, [b] Sritama Mukherjee, [c] Kim Nygård, [d] Zoltán Bacsik,[e] Aji P. Mathew.* [a]

---

[a] Dr. S. Lombardo, H. Khalili, Prof. A.P. Mathew, Department of Chemistry, Stockholm University, 10691 Stockholm, Sweden. E-mail: salvatore.lombardo@su.se; aji.mathew@su.se

[b] Dr S. Yu, Unit of Material, Surface and Barrier, Department of sustainable materials and packaging, RISE Research Institute of Sweden, 22363, Lund, Sweden

[c] Dr. S. Mukherjee, Department of Fiber and Polymer Technology, School of Chemistry, Biotechnology and Health, KTH Royal Institute of Technology, 10044 Stockholm, Sweden.

[d] Dr. K. Nygård, MAX IV Laboratory, Lund University, 22100 Lund, Sweden

[e] Dr. Z. Bacsik, Institute of Chemistry, University of Miskolc, 3515 Miskolc, Hungary

1. **Supporting figures**

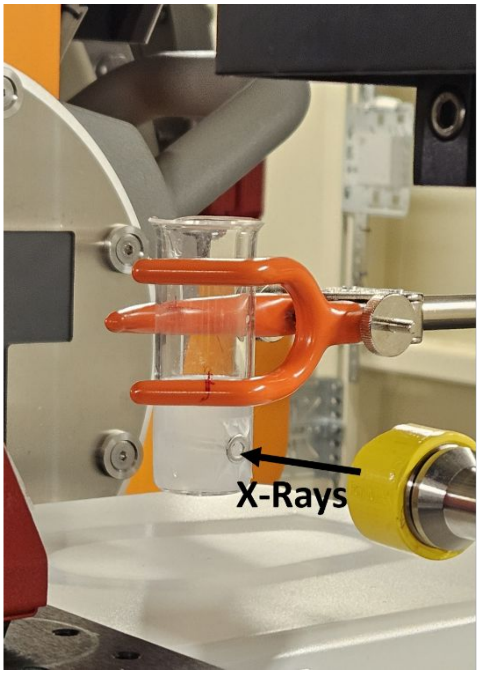

Figure S1. Image of reactor and setup used in our experiments.

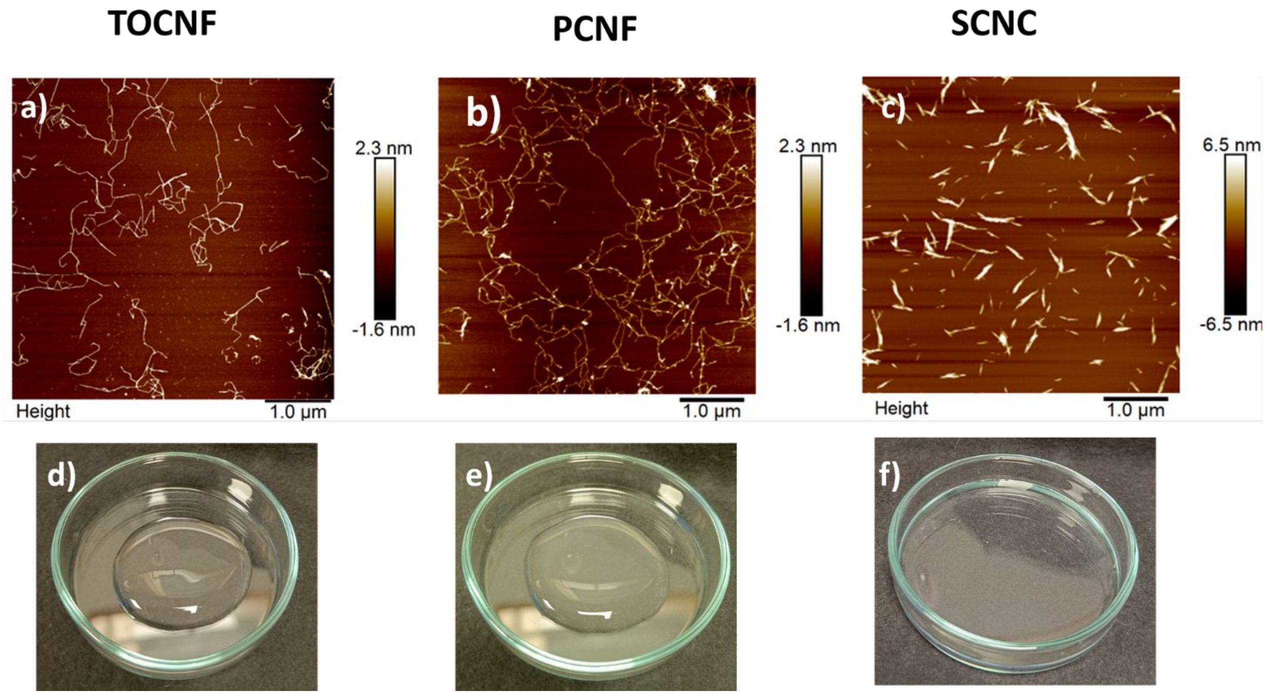

Figure S2. Top: AFM images of TOCNF (a), PCNF (b), and SCNC (c). Down: images of suspensions of TOCNF (d), PCNF (e), and SCNC (f).

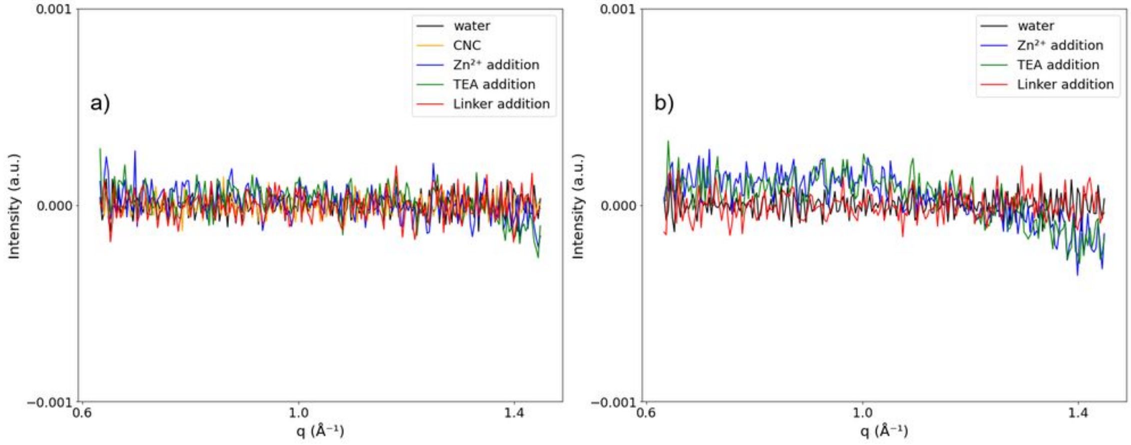

Figure S3. Example of WAXS scattering patterns measured during the full methodology studied after subtraction of the reactor, showing no significant changes. The left plot (a) refers to the synthesis of SCNC/ZIF-8, the right plot (b) shows the synthesis of ZIF-8 in water.

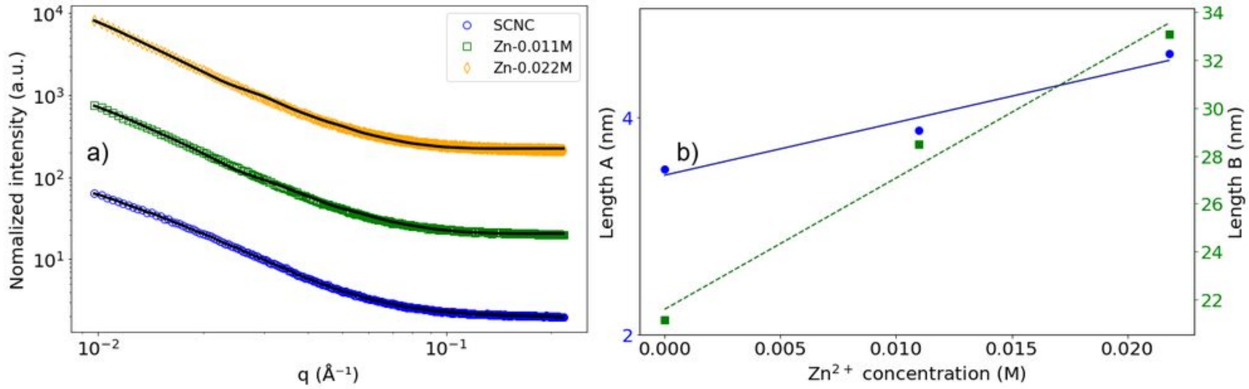

Figure S4. a) Data obtained for SCNC 0.2% (circles), for SCNC in 0.011M $Zn(NO_3)_2$ (squares), and for SCNC in 0.022 M $Zn(NO_3)_2$ (diamonds), and fit using a rectangular prism model. b) Increase of form factor of SCNC after addition of $Zn(NO_3)_2$. SCNC concentration was 0.2 wt%

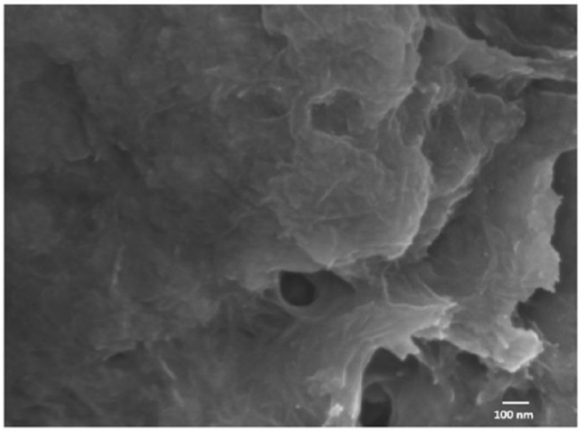

Figure S5. SEM image of the solid material formed after the addition of TEA to SCNC-Zn.

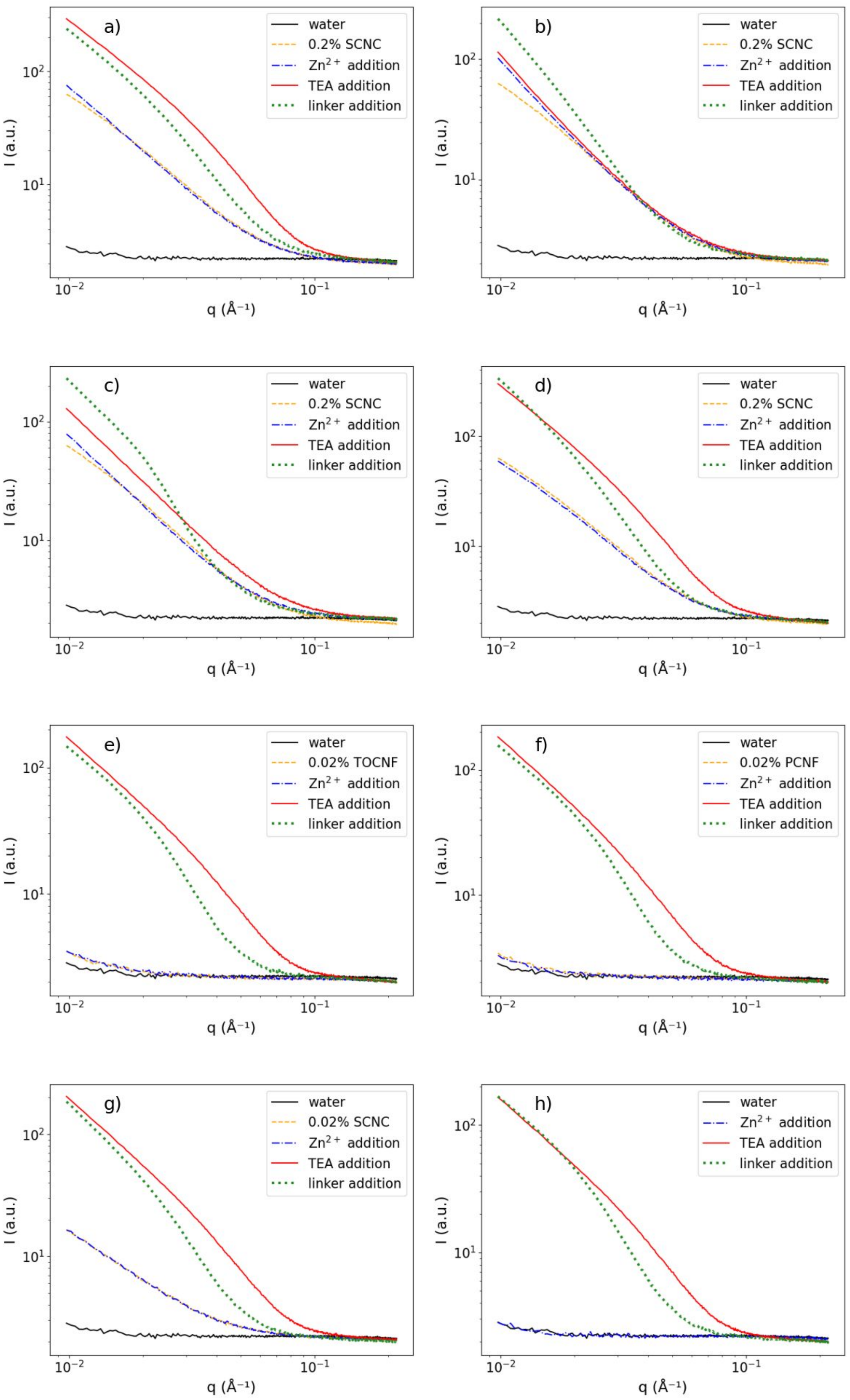

a)
water
0.2% SCNC
Zn^{2+} addition
TEA addition
linker addition
b)
water
0.2% SCNC
Zn^{2+} addition
TEA addition
linker addition
c)
water
0.2% SCNC
Zn^{2+} addition
TEA addition
linker addition
d)
water
0.2% SCNC
Zn^{2+} addition
TEA addition
linker addition
e)
water
0.02% TOCNF
Zn^{2+} addition
TEA addition
linker addition
f)
water
0.02% PCNF
Zn^{2+} addition
TEA addition
linker addition
g)
water
0.02% SCNC
Zn^{2+} addition
TEA addition
linker addition
h)
water
Zn^{2+} addition
TEA addition
linker addition
I (a.u.)
q (Å^{-1})

Figure S6: Example of SAXS scattering patterns measured during the full methodology for synthesis of CelloZIF-8 in water. All datasets used for kinetics modelling are included, the specific conditions for each experiment are given in Table S1, more specifically, panels **a-h** shows experiments E1 – E8 respectively . SCNC at, 0.2 wt% (a-d) , SCNC, TOCNF and PCNF suspensions at 0.02 wt%.(e-g) and h) the control with out the nanocellulose are given. Beaker with water (black solid line), nanocellulose suspension (orange dashed line), addition of $Zn(NO_3)_2$ (blue dash-dotted line) to a nanocellulose suspension, addition of TEA (red solid line), and addition of Hmim (green dotted line) to start the formation the metal organic framework.

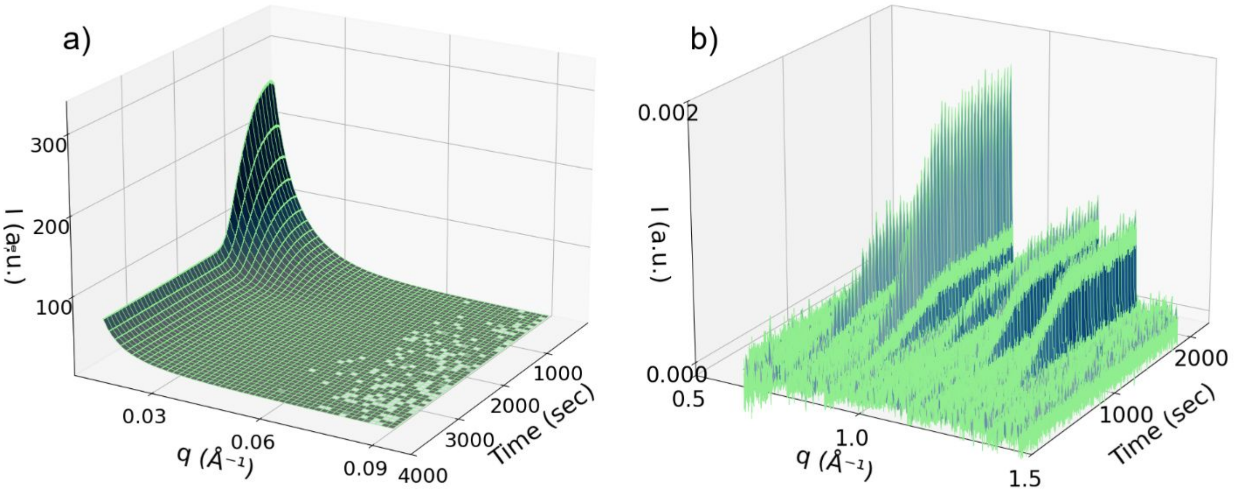

Figure S7. Time-resolved scattering patterns recorded in the SAXS (a) and WAXS region (b) for E1 (Conditions are shown in Table S1).

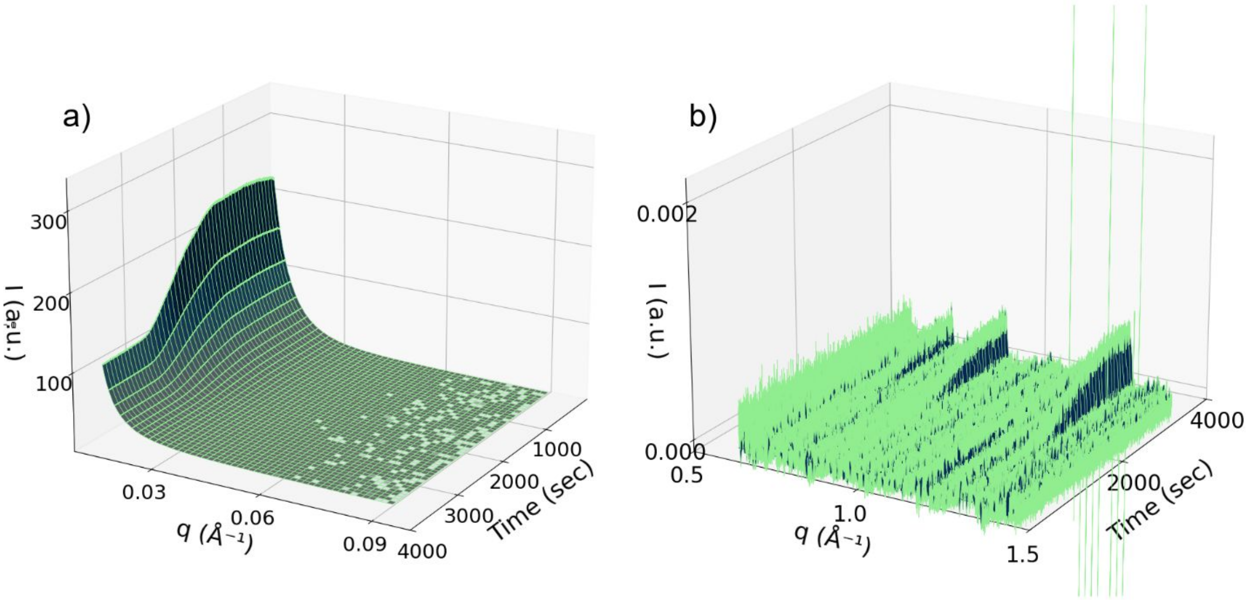

Figure S8. Time-resolved scattering patterns recorded in the SAXS (a) and WAXS region (b) for E2 (Conditions are shown in Table S1).

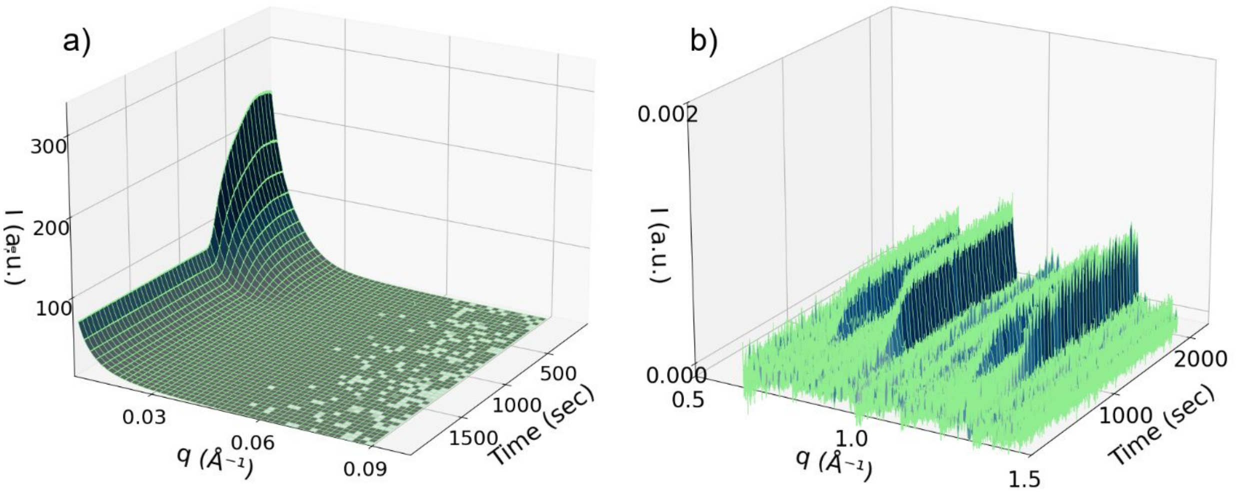

Figure S9. Time-resolved scattering patterns recorded in the SAXS (a) and WAXS region (b) for E3 (Conditions are shown in Table S1).

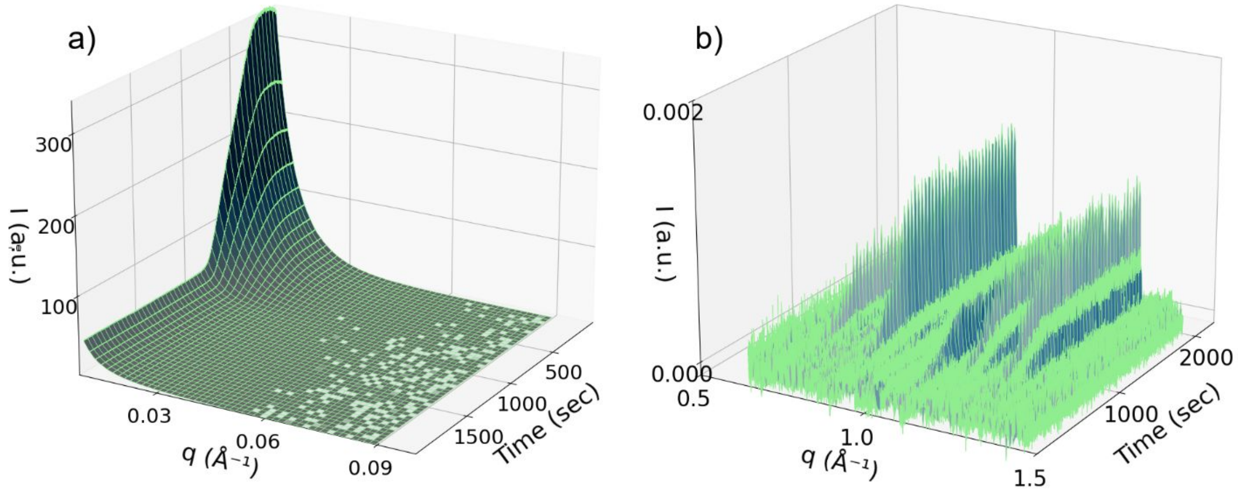

Figure S10. Time-resolved scattering patterns recorded in the SAXS (a) and WAXS region (b) for E4 (Conditions are shown in Table S1).

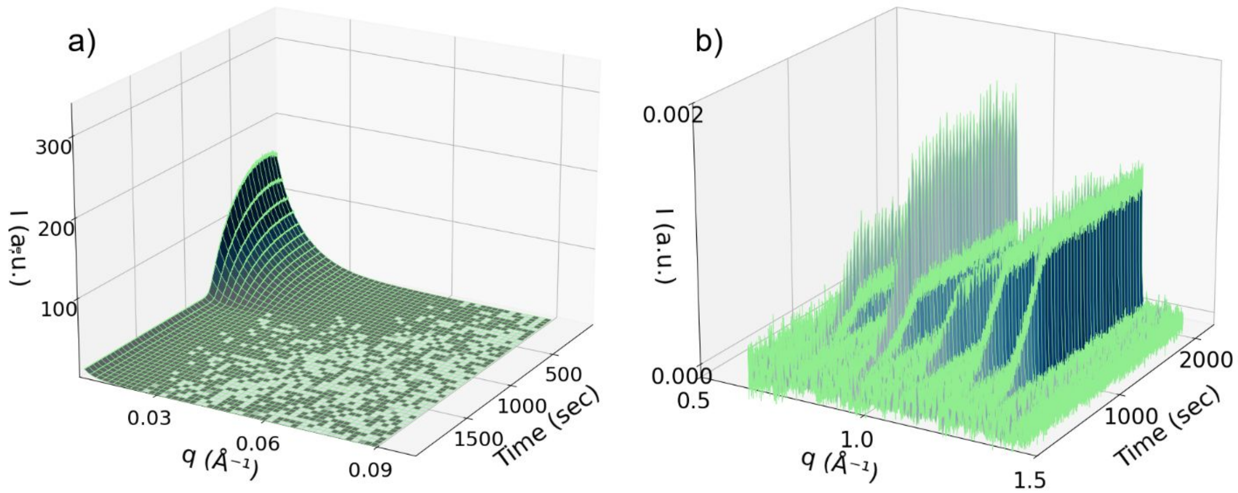

Figure S11. Time-resolved scattering patterns recorded in the SAXS (a) and WAXS region (b) for E5 (Conditions are shown in Table S1).

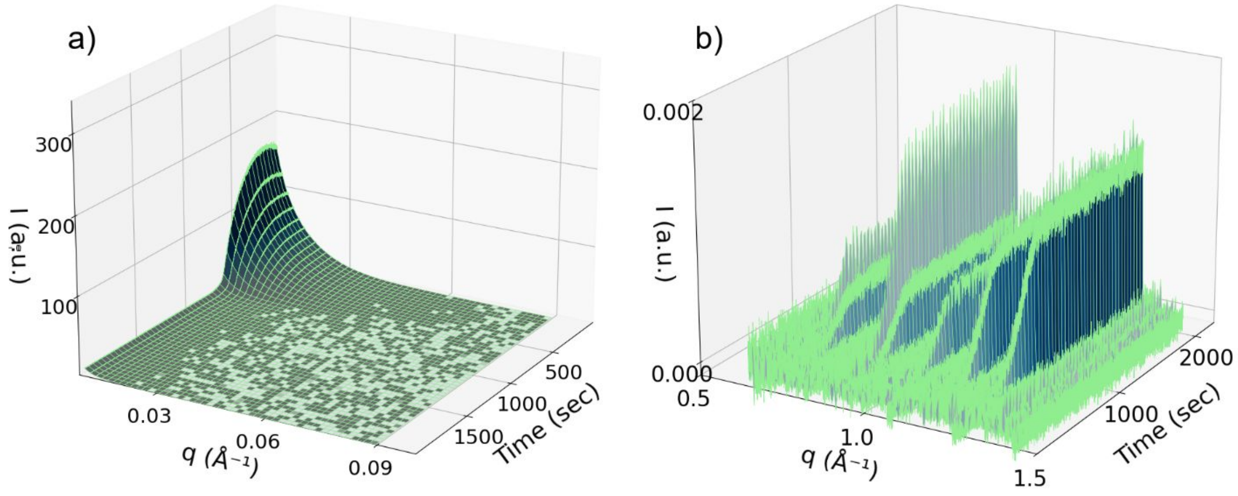

Figure S12. Time-resolved scattering patterns recorded in the SAXS (a) and WAXS region (b) for E6 (Conditions are shown in Table S1).

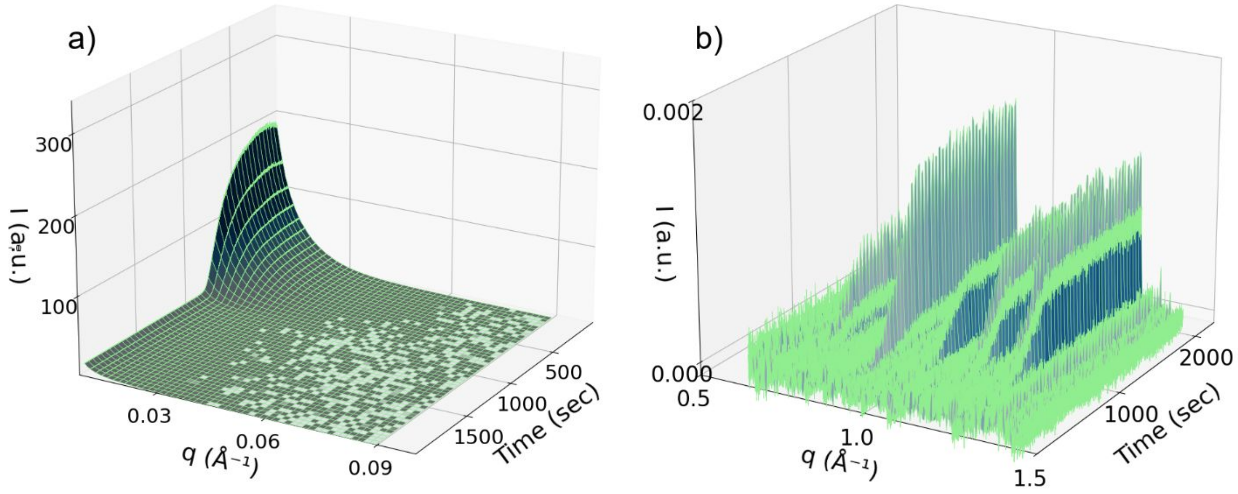

Figure S13. Time-resolved scattering patterns recorded in the SAXS (a) and WAXS region (b) for E7 (Conditions are shown in Table S1).

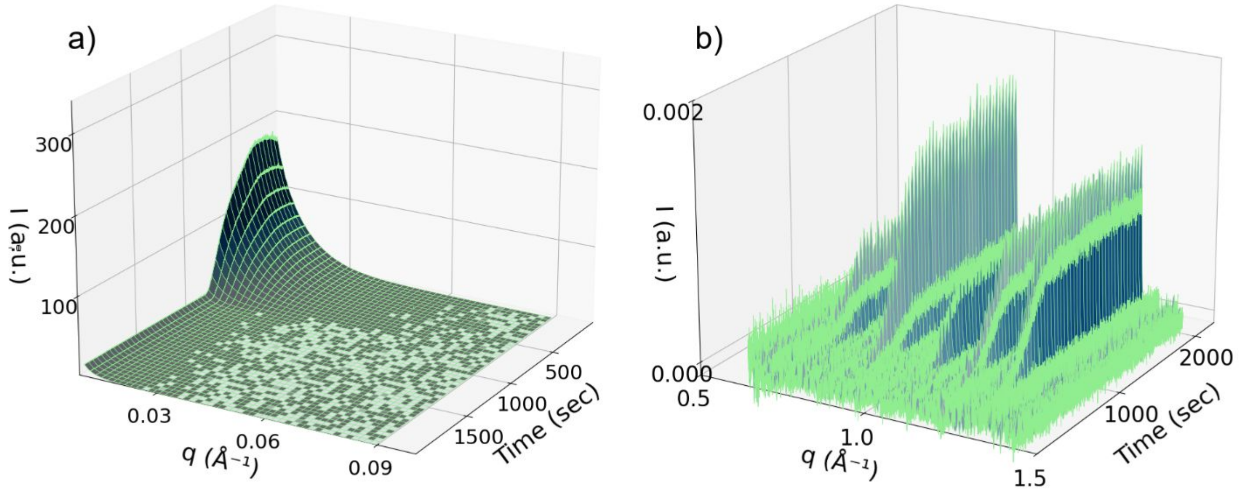

Figure S14. Time-resolved scattering patterns recorded in the SAXS (a) and WAXS region (b) for E8 (Conditions are shown in Table S1).

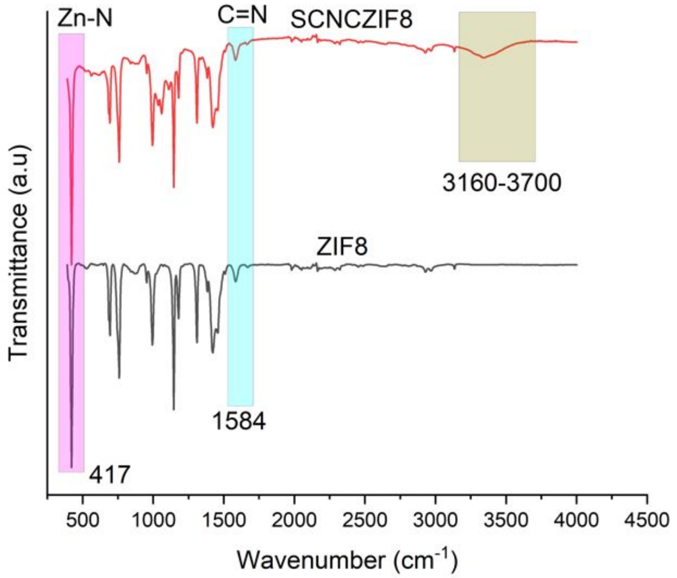

Figure S15. FTIR spectra of SCNC/ZIF-8 and ZIF-8 after purification. The synthesis conditions are the same as reported in Figure 2.

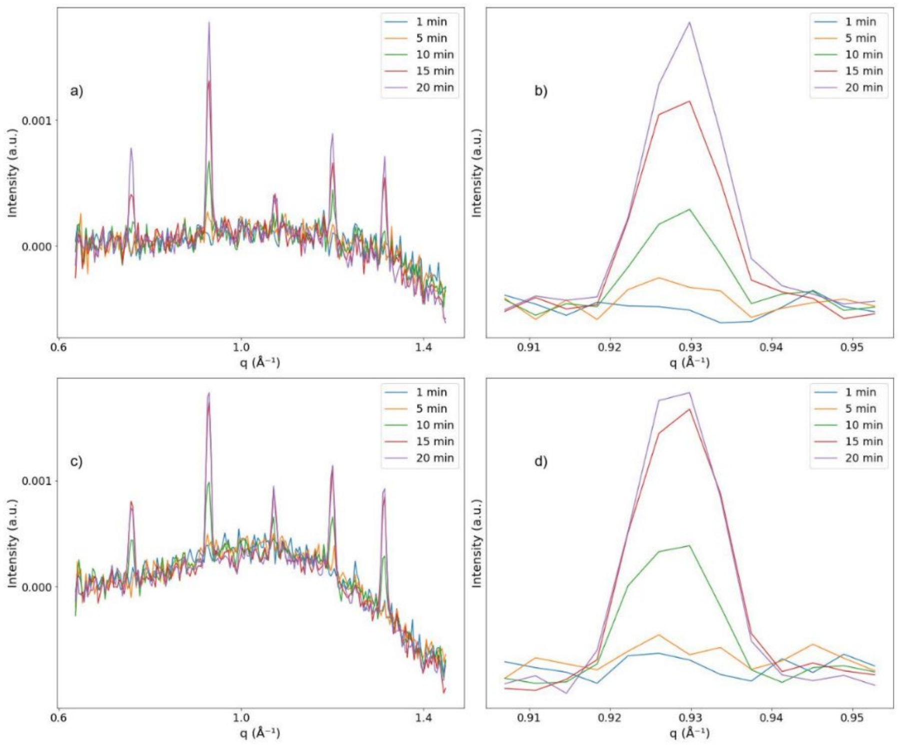

Figure S16. WAXS scattering pattern at selected time for synthesis of CelloZIF-8 at all range used (a), and with focus in the peak of highest intensity (b). Scattering pattern ZIF-8 (c, d) at selected time for synthesis of ZIF-8 in water at all range used (c), and with focus in the peak of highest intensity (d). Conditions for synthesis of CelloZIF-8 and ZIF-8 are reported in Table S1 for E1 and E8 respectively.

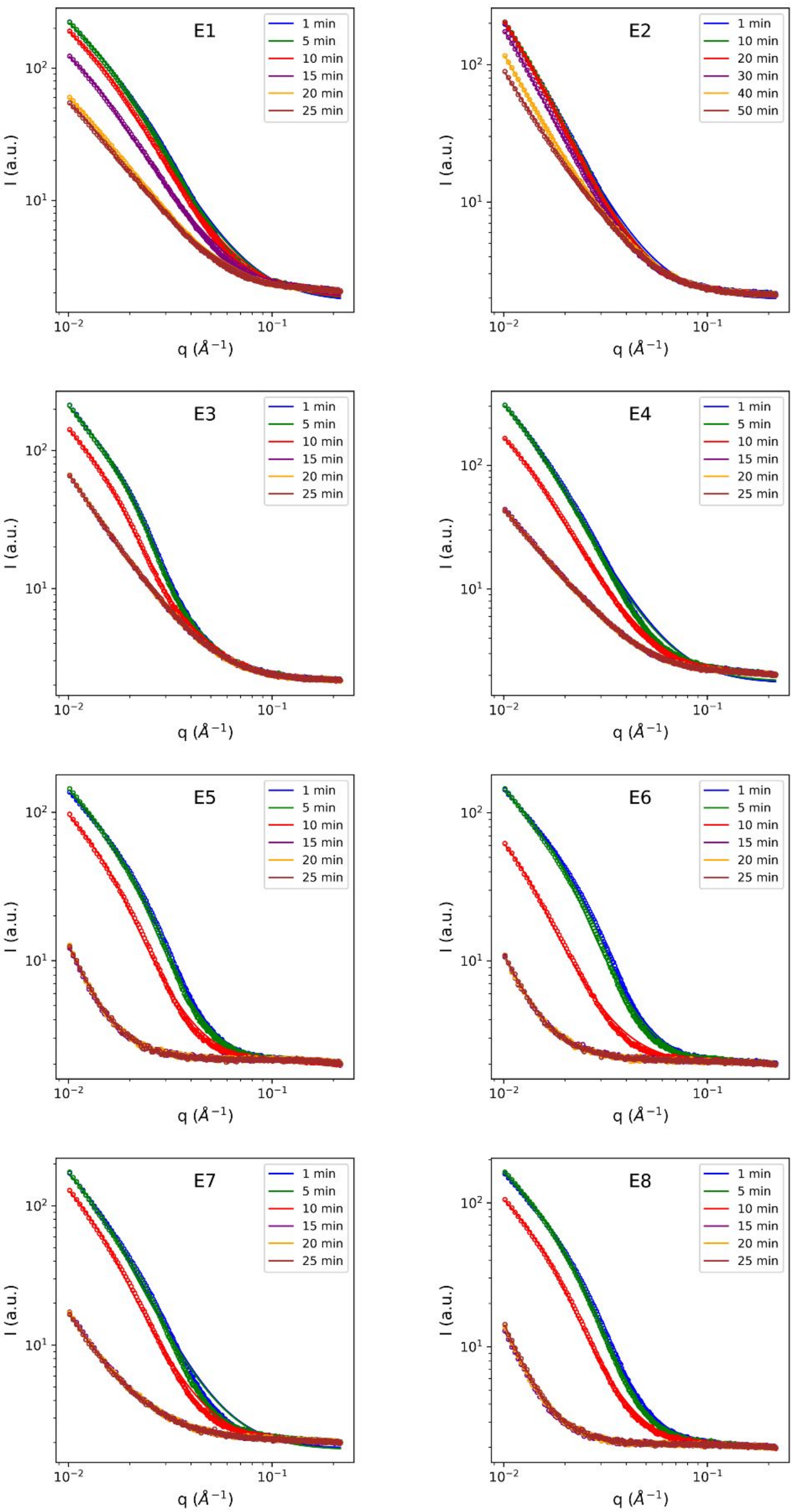

Figure S17. SAXS scattering pattern at selected time at all q range used for experiments. The data shown represents experiments E1-E8 summarised in Table S1. Formulations with SCNC at, 0.2

wt% (E1-E4) , SCNC, TOCNF and PCNF suspensions at 0.02 wt%.(E5-E7) and (E8) the control with out the nanocellulose are given.

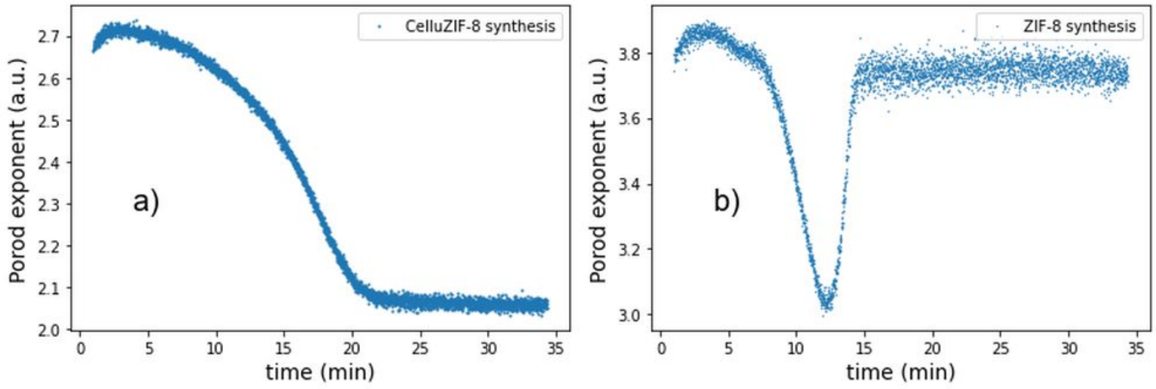

Figure S18. Variation of the Porod exponent from the Guiner-Porod model for synthesis of CelloZIF-8 (a) ZIF-8 (b). Conditions for synthesis of CelloZIF-8 and ZIF-8 are reported in Table S1 for E1 and E8 respectively.

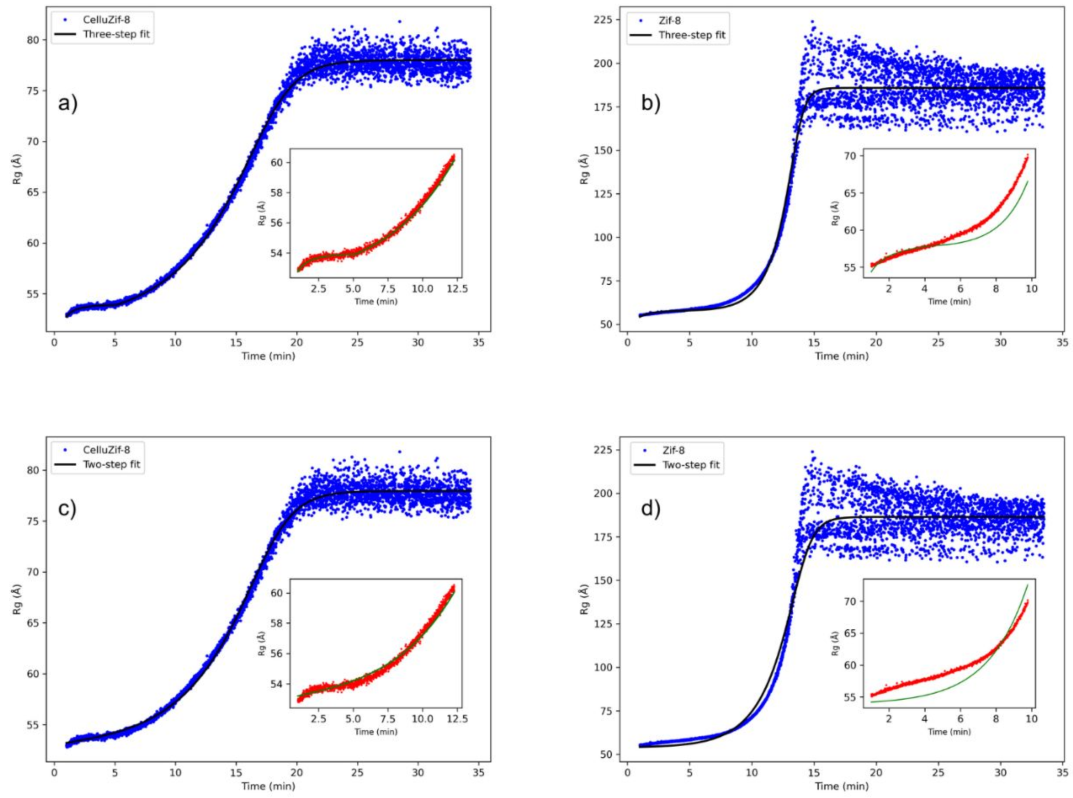

Figure S19. Comparison of the fitting obtained with three-step model for synthesis of CelloZIF-8 (a) and ZIF-8 (b), with the fitting obtained with two-step model for synthesis of CelloZIF-8 (c)

ZIF-8 (d). The insets show the initial part of the curve, where the additional exponential step is applied. Conditions for synthesis of CelloZIF-8 and ZIF-8 are reported in Table S1 for E1 and E8 respectively.

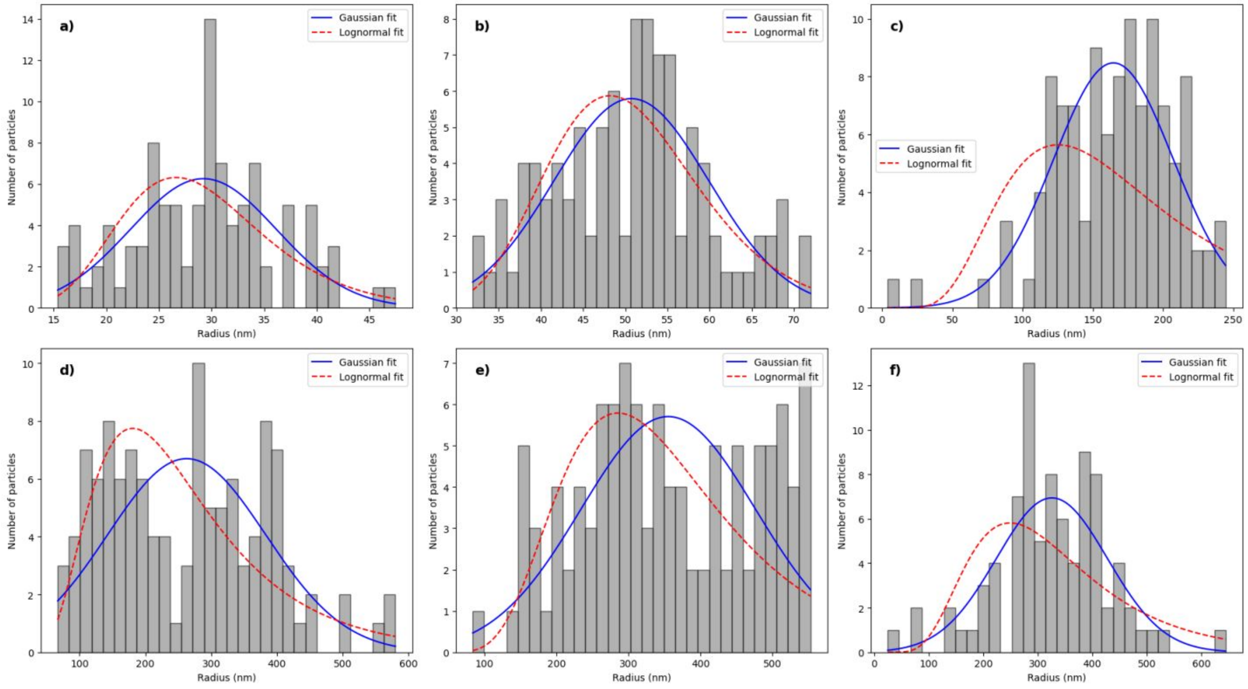

Figure S20. Histograms showing the particle size distributions of CelloZIF and ZIF-8 measured by SEM: (a) SNC/ZIF-8 (E1) at early stage of reaction for particles shown in Figure 4a, and (b) at the end of reaction for particles shown in Figure 4b, (c) SCNC/ZIF-8 (E7) for particle shown in Figure 5b, (d) TOCNF/ZIF-8 (E5) for particle shown in Figure 5c, (e) PCNF/ZIF-8 for particle shown in Figure 5d and (f) ZIF8 for particle shown in Figure 5a.

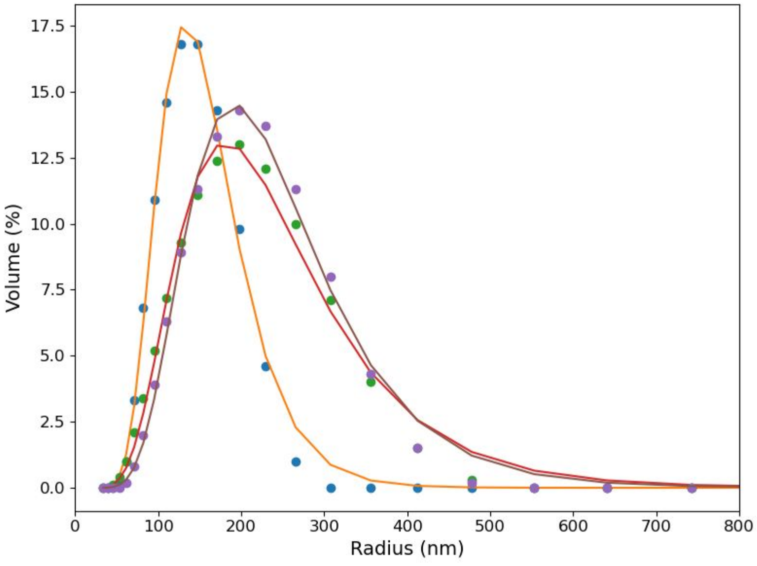

Figure S21. Dynamic light scattering profile and lognormal distribution for pristine ZIF-8. Synthesis conditions were the same as shown in Table S1 for E8. The sample was purified before analysis, as described in the method section.

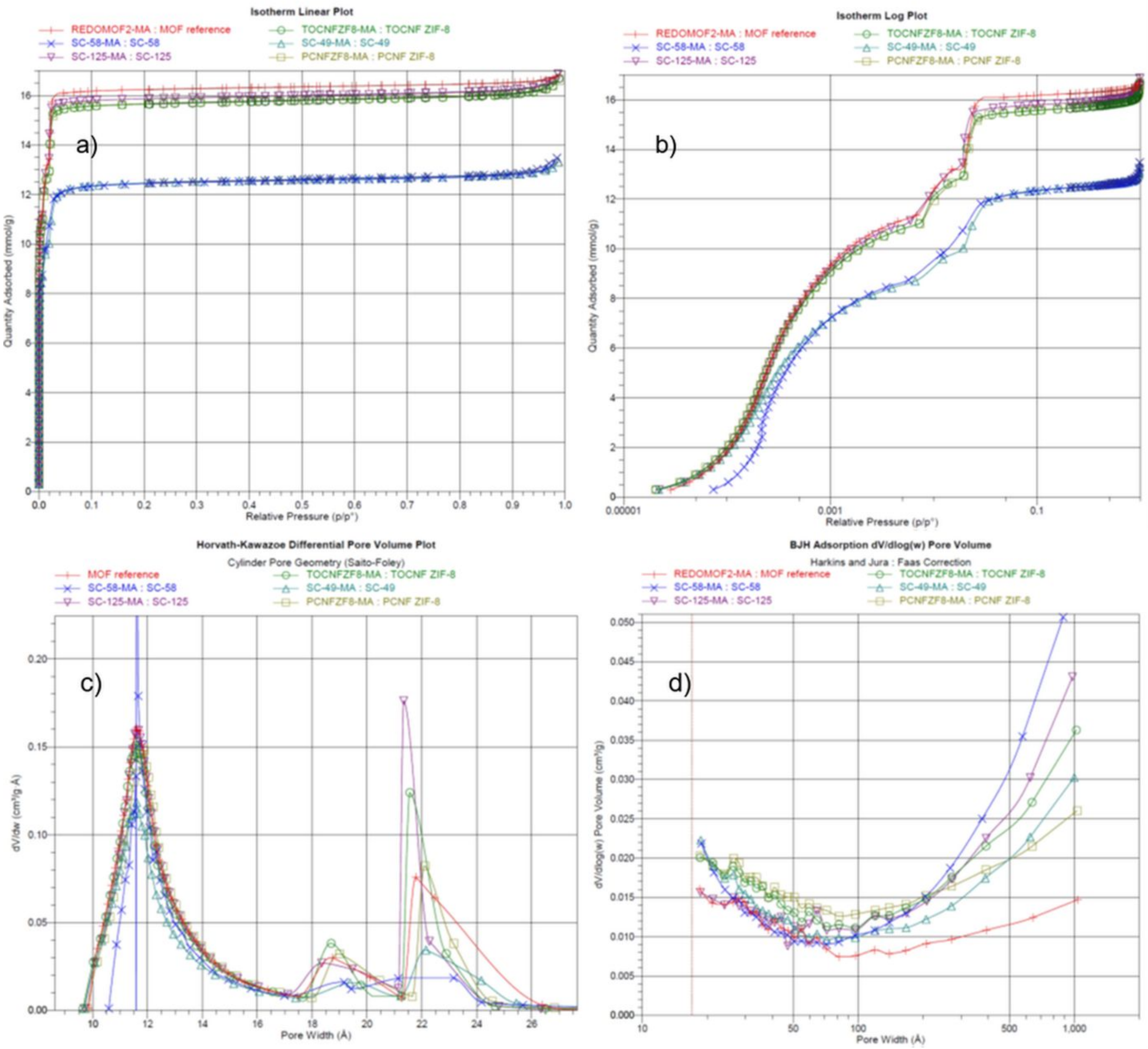

Figure S22. Adsorption isotherms (a-b) and pore size distributions (c-d) of all studied materials shown in Table 1.

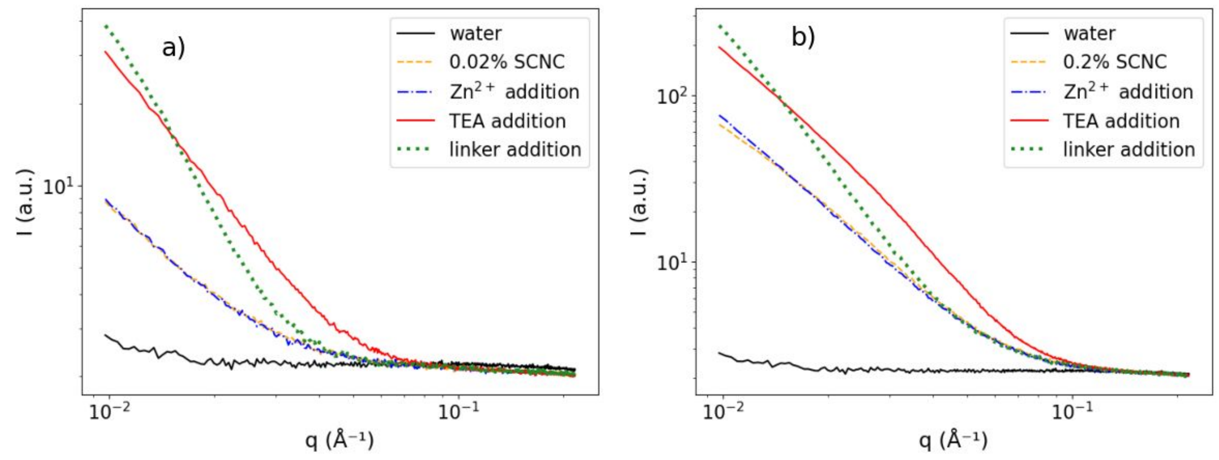

Figure S23. Example of SAXS scattering patterns measured during the full methodology studied, which were not used for data modelling. a) synthesis of SCNF/ZIF-8 adding 0.02 wt% SCNC (orange dashed line), 2 mM $Zn(NO_3)_2$ (blue dash-dotted line), 2 mM TEA (red solid line), and addition of 0.14 M Hmim (green dotted line). b) synthesis of SCNF/ZIF-8 adding 0.2 wt% SCNC (orange dashed line), 1 mM $Zn(NO_3)_2$ (blue dash-dotted line), 1 mM TEA (red solid line), and addition of 0.18 M Hmim (green dotted line).

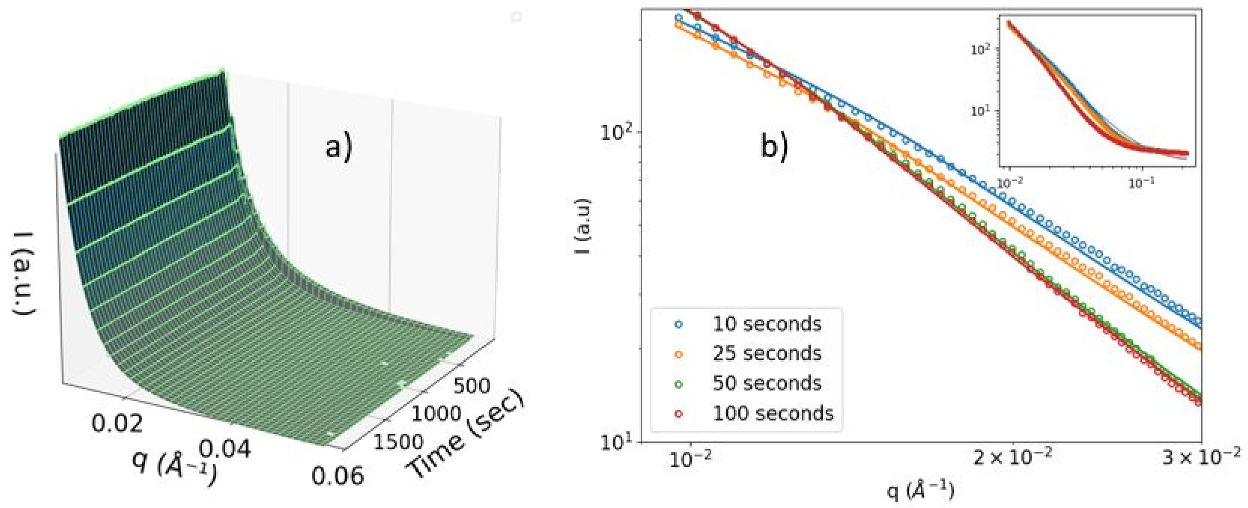

Figure S24. a) SAXS 3d plot, and b) selected scattering data and Guiner-Porod fit showing the porod region, and the full scan (inset) for data shown in figure 6b. The following conditions were used: 15 mL SCNC 0.2%, 0.2 mL $Zn(NO_3)_2$ 0.84 M, 0.025 mL TEA, 1 mL Hmim 3 M.

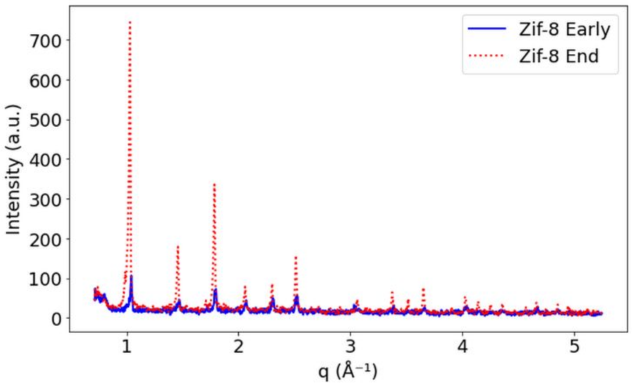

Figure S25. Powder XRD of pristine ZIF-8 obtained after 4 minutes of reaction (blue solid line) and after 1h of reaction (red dotted line), following the conditions shown in Table S1 for E8.

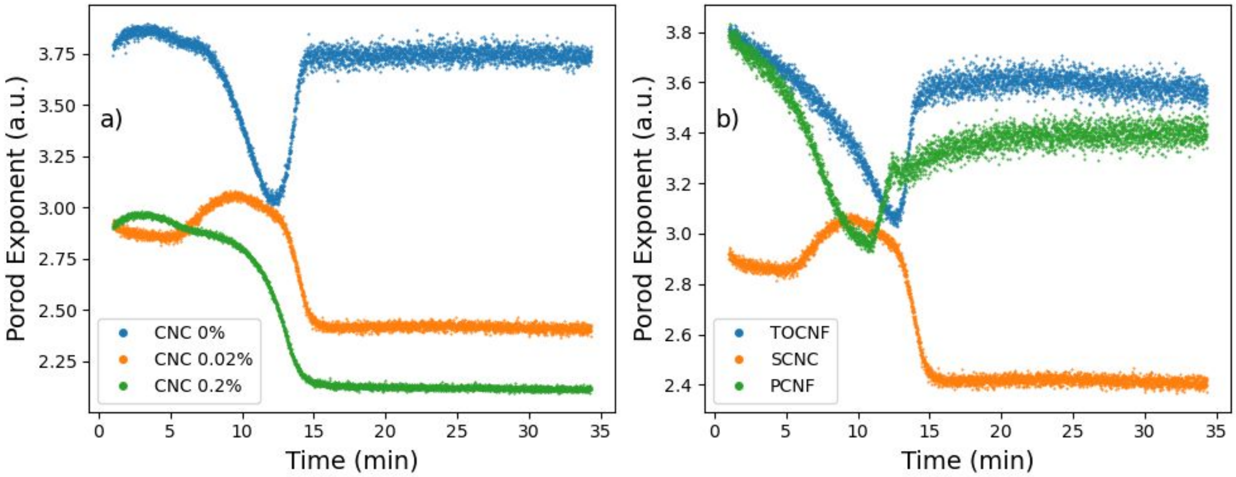

Figure S26. Time evolution of the Porod exponent as function of the SCNC concentration (E4-E7-E8) (a), and for nanocellulose of different surface functionality (E5-E7) (b).

2. Supporting tables

Table S1. List of conditions of the experiments used for fitting.

Name Experiment	$Zn(NO_3)_2$ (mM)	TEA (mM)	ZIF-8/NC Mass Fraction[a]	NC type	SCNC wt%	Conc Hmim (M)	Total Volume (mL)
E1	8.7	9.4	1.3	SCNC	0.2	0.6	19.2
E2	4.7	5.2	0.6	SCNC	0.2	0.3	17.9
E3	14.3	15.3	2.6	SCNC	0.2	1.0	23.9
E4	14.1	15.0	2.6	SCNC	0.2	1.0	23.9

E5	14.1	15.0	25.7	TOCNF	0.02	1.0	23.9
E6	14.1	15.0	25.7	PCNF	0.02	1.0	23.9
E7	14.1	15.0	25.7	SCNC	0.02	1.0	23.9
E8	14.1	15.0	100	No SCNC	-	1.0	23.9

[a]The mass of ZIF-8 was determined theoretically from stoichiometry, assuming complete conversion of Zn^{2+}. For calculations we used molar mass of ZIF-8 of 227.6 g mol^{-1}.

Table S2. Avrami constants determined from WAXS data, and final particle radius determined by the Scherrer equation.

Name Experiment	k_a (min^{-1})	R^2	R_f (Å)	k_a (min^{-1}) [a]	R^2 [a]
E1	3.27E-04 ± 1E-06	0.99	51 ± 1	3.42E-04 ± 2E-06	0.98
E2	1.81E0-5 ± 1E-07	0.88	35 ± 4	1.93E0-5 ± 3E-07	0.67
E3	5.74E-04± 6E-06	0.91	34 ± 3	6.7E-04 ± 1E-05	0.78
E4	8.71E-04 ± 4E-06	0.98	45 ± 2	8.52E-04 ± 7E-06	0.95
E5	7.11 E0-4 ± 3E-06	0.99	47 ± 1	7.11 E0-4 ± 3E-06	0.96
E6	1.045 E-03 ± 5.E-06	0.99	47 ± 1	1.048 E-03 ± 8E-06	0.97
E7	5.92 E-04 ± 2E-06	0.99	45 ± 2	5.90 E-04 ± 4E-06	0.97
E8	6.50E-04 ± 2E-06	0.99	43 ± 1	6.36E-04 ± 4E-06	0.97

[a]Avrami constants were determined from the 002 crystallization peak

Table S3. Fitting values from SAXS data, applying a two-step model.

Name Experiment	k_1 (min^{-1})	k_2 (M^{-1} min^{-1})	R_1 (Å)	R_f (Å)	R^2
E1	4.3E-06 ± 3E-07	48.1 ± 0.3	52.75 ± 0.05	77.54 ±0.01	0.99

E2	1.3E-08 ± 3E-09	31.6 ± 0.3	86.2 ± 0.2	121.2 ± 0.1	0.91
E3	1.1E-08 ± 3E-09	111 ± 1	61.7 ± 0.2	105.1 ± 0.1	0.96
E4	1.1E-08 ± 4E-09	104 ± 1	66.8 ± 0.2	99.6 ± 0.1	0.93
E5	1.5E-08 ± 4E-09	92 ± 2	56.0 ± 0.5	187.9 ± 0.3	0.95
E6	1.54E-08 ±4E-09	111 ± 1	53.6 ± 0.5	180.0 ± 0.3	0.96
E7	6E-09±1E-09	98.4 ± 0.6	65.1 ± 0.2	139.5 ± 0.1	0.97
E8	9E-09±2E-09	95.8 ± 0.7	53.0 ± 0.4	187.4 ± 0.2	0.98

Table S4. Fitting values from SAXS data, applying a three-step model.

Name Experiment	k_1 (min^{-1})	k_2 (min^{-1})	k_3 (M^{-1} min^{-1})	R_1 (Å)	R_f (Å)	R^2
E1	3.76 ± 0.02	9.3E-06 ± 4E-07	70.2 ± 0.3	54.03 ± 0.01	77.54 ± 0.01	0.99
E2	3.38 ± 0.02	2.4E-08 ± 4E-09	50.2 ± 0.7	85.16 ± 0.03	119.2 ± 0.1	0.91
E3	3.93 ± 0.02	5E-16 ± 4E-16	220 ± 5	63.91 ± 0.01	104.82 ± 0.05	0.93
E4	2.96 ± 0.01	2E-11 ± 1.08E-11	142 ± 4	68.77 ± 0.02	96.71 ± 0.04	0.98
E5	2.32 ± 0.01	5E-13 ± 2E-13	151 ± 2	61.87 ± 0.04	173.4 ± 0.1	0.99
E6	2.78 ± 0.02	1.6E-11 ± 4E-12	158 ± 2	58.22 ± 0.03	175.2 ± 0.2	0.98
E7	2.50 ± 0.01	5E-12 ± 2E-12	138 ± 2	68.77 ± 0.03	138.6 ± 0.1	0.98
E8	2.61 ± 0.02	2.4E-12 ± 7E-13	141± 2	58.67 ± 0.04	182.9 ± 0.2	0.94
